# Supplementary figures and images for: Myotis rufoniger genome sequence and analyses: M. rufoniger’s genomic feature and the decreasing effective population size of Myotis bats
Source: PLoS One. 2017 Jul 5;12(7):e0180418. doi: 10.1371/journal.pone.0180418 (PMC5498047; doi:10.1371/journal.pone.0180418)

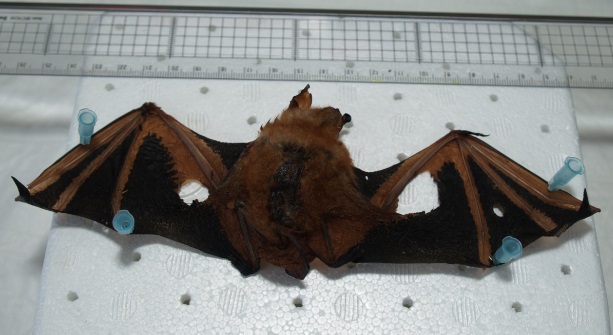

Supplement: S1 Picture — (JPG) [file pone.0180418.s001.jpg]

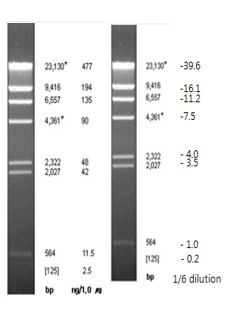

Supplement: S2 Picture — (JPG) [file pone.0180418.s002.jpg]

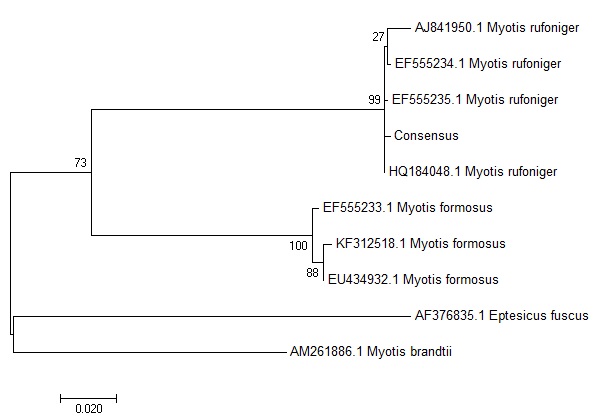

Supplement: S1 Fig — The phylogenetic relationship of Myotis bats was inferred from the alignment of mitochondrial cytochrome b coding sequences. The percentage of trees in which the associated taxa clustered together is shown next to the branches. Each node has its species name and GenBank accession number. (JPG) [file pone.0180418.s003.jpg]

**17-mer estimation of genome size**

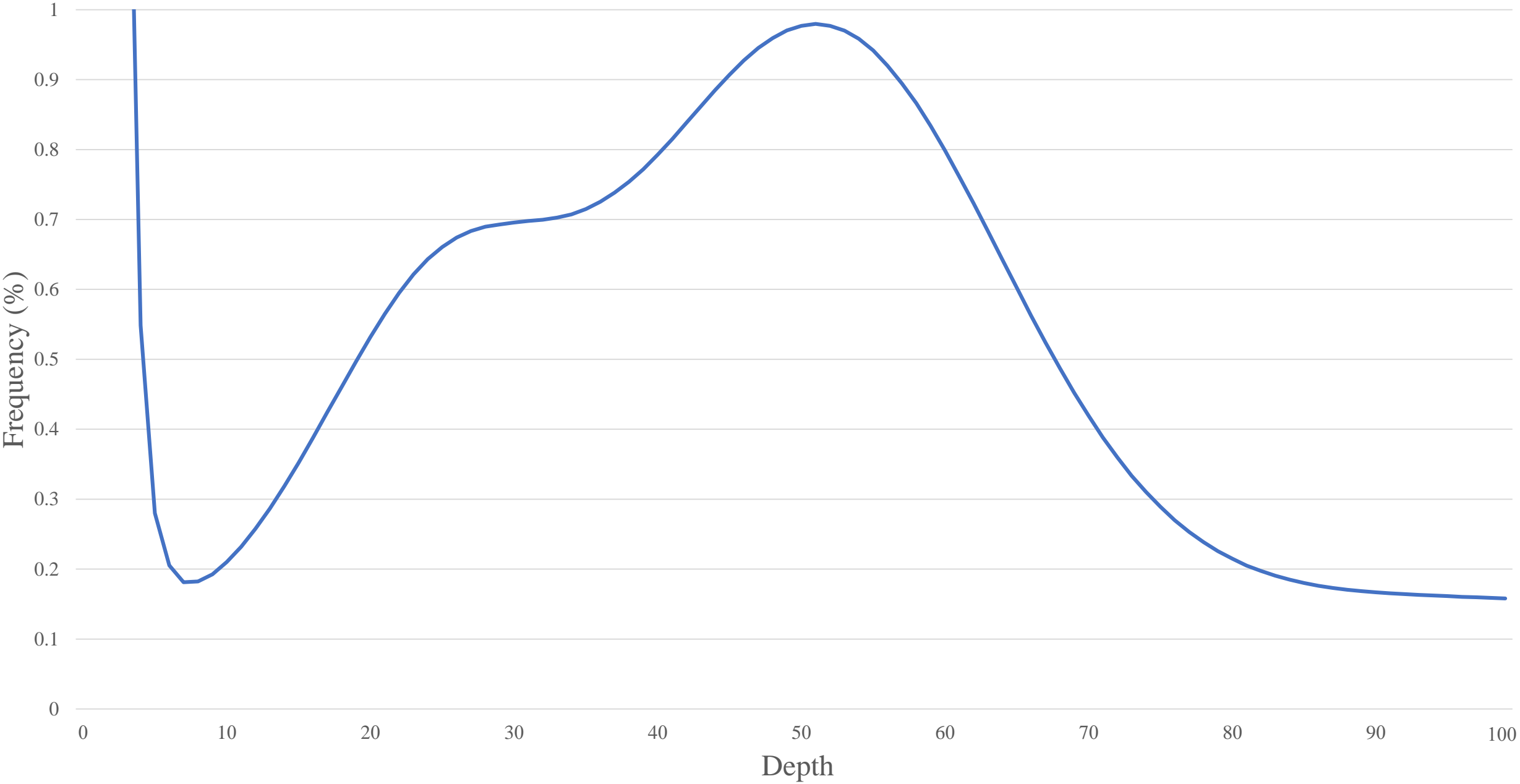

Supplement: S2 Fig — The x-axis represents depth, and the y-axis represents proportion, as calculated by the frequency at a given depth divided by the total frequency at all depths. (PDF) [file pone.0180418.s004.pdf]

(A)

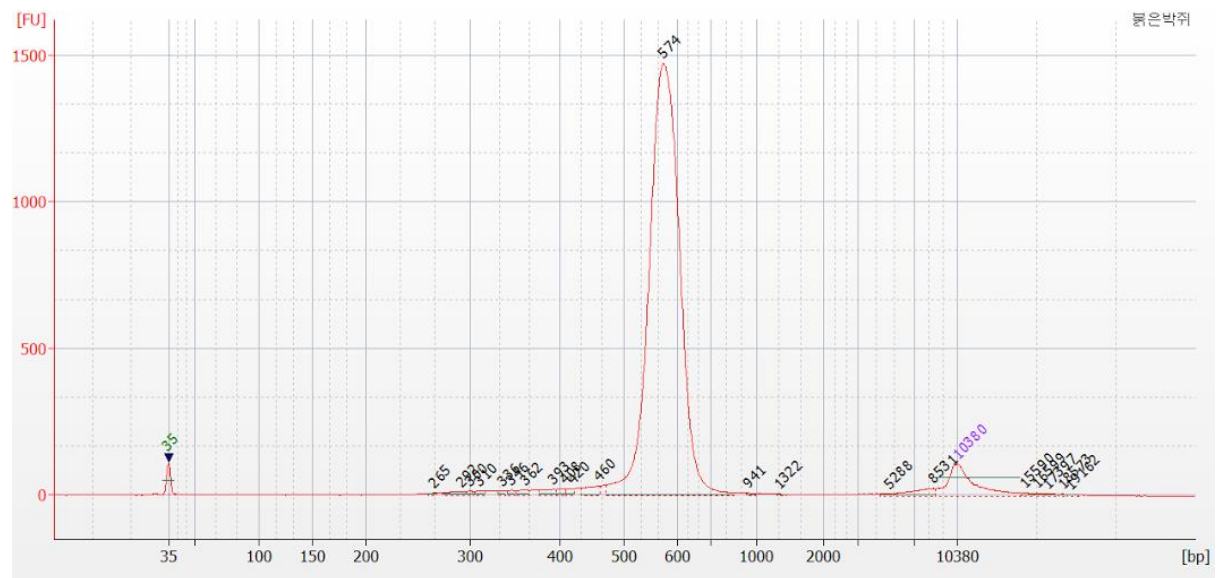

(B)

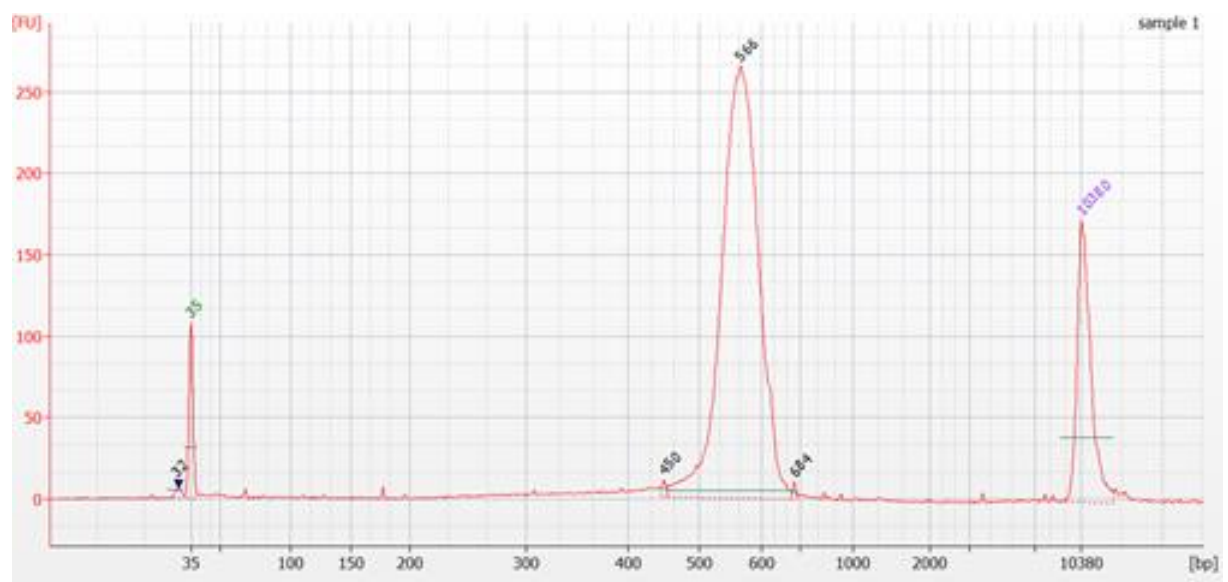

Supplement: S6 Fig — (A) Sequencing library 574bp QC; (B) Sequencing library 566bp QC. (PDF) [file pone.0180418.s008.pdf]
