# Supplementary material for: Myotis rufoniger genome sequence and analyses: M. rufoniger’s genomic feature and the decreasing effective population size of Myotis bats
Source: PLoS One. 2017 Jul 5;12(7):e0180418. doi: 10.1371/journal.pone.0180418 (PMC5498047; doi:10.1371/journal.pone.0180418)
Supplement: S3 Fig — Previously reported bats’ unique amino acid sequence changes within FSHB, GHR, IGF1R, TP53, and MDM2 are highlighted in yellow; (A), Alignment of FSHB encoded peptide sequences; (B), Alignment of GHR-encoded peptide sequences; (C), Alignment of IGF1R encoded peptide sequences; (D), Alignment of TP53-encoded peptide sequences; (E), Alignment of MDM2 encoded peptide sequences; (F), Alignment of SLC45A2-encoded peptide sequences; (G), Alignment of RGS7BP-encoded peptide sequences; (H), Alignment of RHO-encoded peptide sequences; (I), Alignment of OPN1SW-encoded peptide sequences; (J), Alignment of CNGB3-encoded peptide sequences. (PDF) [file pone.0180418.s005.pdf]

(A)

FSHB

M. rufoniger	RVPGCAHHADSXYMYPVATECHCGKCNRDSTDCTVQGLGPSYCSFSEIKE	129
M. davidii	RVPGCAHHADSVYTYPIATECHCGKCNRDSTDCTVQGLGPSYCSFSEIKE	
M. brandtii	RVPGCAHHADSIYTYPVATECHCGKCNRDSTDCTVQGLGPSYCSFSEIKE	
M. lucifugus	RVPGCAHHADSVYTYPVATECHCGKCNRDSTDCTVQGLGPSYCSFSEIKE	
E. fuscus	RVPGCAHQADSVYTYPVATECHCGKCNRDSTDCTVQGLGPSYCSFSEIKE	
P. alecto	KVPGCAHHANSLYAYPVATECHCGKCDSDNTDCTVRGLGPSYCSFSEMKE	
P. vampyrus	KVPGCAHHANSLYAYPVATECHCGKCDSDNTDCTVRGLGPSYCSFSEMKE	
R. aegyptiacus	KVPGCAHHANSLYAYPVATECHCGKCDSDNTDCTVRGLGPSYCSFSEMKE	
B. taurus	KVPGCAHHADSLYTYPVATECHCSKCDSDSTDCTVRGLGPSYCSFREIKE	
E. caballus	KVPGCAHHADSLYTYPVATACHCGKCNSDSTDCTVRGLGPSYCSFGDMKE	
M. musculus	RLPGCARHSDSLYTYPVATECHCGKCDSDSTDCTVRGLGPSYCSFSEMKE	
H. glaber	RVPGCAHHADSLYTYPVATKCHCGKCDSDSTDCTVRGLGPSYCSFSEMA-	
H. sapiens	RVPGCAHHADSLYTYPVATQCHCGKCDSDSTDCTVRGLGPSYCSFGEMKE	
M. domestica	SLPGCANQADSLYSYPVATACHCGSCDTDSTDCTVRGLGPSYCSFNERKE	

(B)

GHR

M. rufoniger	QMSPVPCEEDFRFPWFLIIIFGMFGLTVI-FLFMFSKQQRIKMLILPPVP	301
M. davidii	QMSPVPCEEDFRFPWFLIVIFGMFGLTVI-FLFMFSKQQRIKMLILPPVP	
M. brandtii	QMSPVPCEEDFRFPWFLIIIFGMFGLTVI-FLFVFSKQQRIKMLLLPPVP	
M. lucifugus	YEILFSCVSDFRFPWFLIIIFGMFGLTVI-FLFMFSKQQRIKMLLLPPVP	
E. fuscus	QMSPVPCEEDFQFPWFLIIIFGMFGLTVI-FLFMFSKQQRIKMLILPPVP	
P. alecto	QMSPLACEEDFRFPWFLIIIFGIFGLTVILFLFIFSKQQRIKMLILPPVP	
P. vampyrus	QMSPLACEEDFRFPWFLIIIVGIFGLTVILFLFIFSKQQRIKMLILPPVP	
R. aegyptiacus	QMSPLACEEDFRFPWFLIIIFGIFGLTVILFLFIFSKQQRIKMLILPPVP	
B. taurus	QMNPSACEEDFQFPWFLIIIFGILGLAVTLYLLIFSKQQRIKMLILPPVP	
E. caballus	QMSPLACEEDFQFPWFLIIILGIFGLTVILFLFIFSKQQRIKMLILPPVP	
M. musculus	--ILEACEEDIQFPWFLIIIFGIFGVAVMLFVVIFSKQQRIKMLILPPVP	
H. glaber	QLSSFTCEEEFQFPWFLIMIFGIFGLMVMLLVVIFSKQQRIKMLILPPVP	
H. sapiens	QMSQFTCEEDFYFPWLLIIIFGIFGLTVMLFVFLFSKQQRIKMLILPPVP	
M. domestica	S-ECEEKINDFRFPWFLIIIFGILGLTVVLFVFILSKQQRIKLLILPPVP	

(C)

IGF1R

M. rufoniger	AAYDSHAHLVIALPIVIVLTVLGLALTLYIIHRKR--NRMGNGVLY	974
M. davidii	AAYDSHAHLVIALPIAIVLTVLGLALTLYIIHRKR--NRMGNGVLY	
M. brandtii	AAYDSHAHLVIALPIAIVLTVLGLALTLYVIHRKR--NRMGNGVLY	
M. lucifugus	AAFDSHAHLVIALPIAIVLTVLGLALTLYIIHRKR--NRMGNGVLY	
E. fuscus	AAMDNYAHLVIALPIAIVLIVCGLAVTLYIIHRKR--NRMGNGVLY	
P. alecto	TTYENFMHLIIALPIAILLIVGGLVIMLYVFHRKRNSNRLGNGVLY	
P. vampyrus	TTYENFMHLIIALPIAILLIVGGLVIMLYVFHRKRNSNRLGNGVLY	
R. aegyptiacus	ATYENFTHLVIALPVAILLIVAGLVIMLYVFHRKRNSNRLGNGVLY	
B. taurus	TTYENFIHLMIALPIAVLLIVGGLVIMLYVFHRKRNSSRLGNGVLY	
E. caballus	TTYENFIHLIIALPVAVLLIVGGLVIMLYVFHRKRNNSRLGNGVLY	
M. musculus	TTYENFMHLIIALPVAILLIVGGLVIMLYVFHRKRNNSRLGNGVLY	
H. glaber	TTYENFIHLIIALPVAILLIVGGLVIMLYVFHRKRNNSRLGNGVLY	
H. sapiens	TGYENFIHLIIALPVAVLLIVGGLVIMLYVFHRKRNNSRLGNGVLY	
M. domestica	QDYENFLHLMIALPIAGLFIVGGLLIMWYVFNKKRNSDRLGNGVLY	

(D)

TP53

M. rufoniger	ALPTNTDSSPP-PKXPVDXEXFTLQIRGRERFETFRKLNE	378
M. davidii	ALPTNTDSSPP-PKKPADEEYFTLQIRGRERFETFRKLNE	
M. brandtii	ALPTNTDPSPP-PKKPADEEYFTLQIRGRERFETFRKLNE	
M. lucifugus	ALPTNTDPSPP-PKKPADEEYFTLQIRGRERFETFRKLNE	
E. fuscus	ALPTDTDSSPP-PKMPVDGEYFTLQIRGRARFEMFRELNE	
P. alecto	ALPTDTTSSSPSPKMPLDEEYFTLQIRGRKNFEILRELNE	
P. vampyrus	ALPTDTTSSSPSPKMPLDEEYFTLQIRGRKNFEILRELKE	
R. aegyptiacus	ALPTNTTSSSPSPKMPLDEEYFTLQIRGRKNFEMLRELNE	
B. taurus	ALPTNTSSSPQPKKKPLDGEYFTLQIRGFKRYEMFRELND	
E. caballus	VLSSNTSSSPPQKKKPLDGEYFTLQIRGRERFEMFRELNE	
M. musculus	ALPTCTSASPPQKKKPLDGEYFTLKIRGRKRFEMFRELNE	
H. glaber	ALPTGTNSSPQPKKKPLDGEYFTLKIRGRERFEMFRELNE	
H. sapiens	ALPNNTSSSPQPKKKPLDGEYFTLQIRGRERFEMFRELNE	
M. domestica	ALPTTPGSTPKAKKKLVEGEYFTLQIRGRQRYELLREINE	

(E)

MDM2

M. rufoniger	ENSDELSGERQRKRHRSDSISFSFDESLALCVIREIRCER	210
M. davidii	ENSDELSGERQRKRHRSDSISFSFDESLALCVIREIRCER	
M. brandtii	ENSDELSGERQRKRHRSDSISFSFDESLALCVIREIRCER	
M. lucifugus	ENSDELSGERQRKRHRSDSISFSFDESLALCVIREIRCER	
E. fuscus	GNSDELSCERQRKRHRSDSISFSFDESLALCVIREIRCER	
P. alecto	DNSDELPGERQRKRHKSDSISFSFDESLALCVIREIRCER	
P. vampyrus	DNSDELPGERQRKRHKSDSISFSFDESLALCVIREIRCER	
R. aegyptiacus	DNSDELPGERQRKRHKSDSISFSFDENLALCVIREIRCER	
B. taurus	ENSDELPGERQRKRHKSDNISLSFDESLALCVIREICCER	
E. caballus	ENSDELPGERQRKRHKSDSVSLSFDEGLALCVIREICCDG	
M. musculus	ENTDELPGERHRKRRR----SLSFDPSLGLCELREMCSGG	
H. glaber	ENSDELSGERQRKRHKSDSISLSFDESLALCVIRELCCER	
H. sapiens	ENSDELSGERQRKRHKSDSISLSFDESLALCVIREICCER	
M. domestica	ENLDELPGERHRKRHKSDSISLPFDESCALCVISEICCER	

(F)

SLC45A2

M. rufoniger	YRCLCISHLIGWTAFLSNMLFFTDFMGRIVYHGNPYSAHNSTEFLIYERG	214
M. davidii	YRCLCISHLIGWTAFLSNMLFFTDFMGRIVYHGNPYSAHNSTEFLIYERG	
M. brandtii	YRCLCISHLIGWTAFLSNMLFFTDFMGRIVYHGNPYSAHNSTEFLIYERG	
M. lucifugus	YRCLCISHLIGWTAFLSNMLFFTDFMGRIVYHGNPYSAHNSTEFLIYERG	
E. fuscus	YRCLCISHLIGWTAFLSNMLFFTDFMGRIVYHGNPYSAHNSTEFLIYERG	
P. alecto	HRCLCISHLIGWTAFLCNMLFFTDFMGQIVYHGDPYSAHNSTEFLIYERG	
P. vampyrus	YRYLCISHLIGWTAFLCSMLFFTDFMGQIVYHGDPYSAHNSTEFLIYERG	
R. aegyptiacus	YRCLCISHLIGWTAFLSNMLFFTDFMGQIVYHGDPYSAHNSTEFLIYERG	
B. taurus	YRCLCISHLIGWTAFLSNMLFFTDFMGQIVYHGDPYGAHNSTEFLIYQRG	
E. caballus	YRYLCISHLLGWTAFLSNMLFFTDFMGQIVYHGDPYSAHNSTEFLIYQRG	
M. musculus	YRCLCVSHLIGWTAFLSNMLFFTDFMGQIVYHGDPYGAHNSTEFLIYERG	
H. glaber	YRCLCISHLIGWTAFLSNMLFFTDFMGQIVYRGDPYSAHNSTEFLIYERG	
H. sapiens	YRYLCISHLIGWTAFLSNMLFFTDFMGQIVYRGDPYSAHNSTEFLIYERG	
M. domestica	YRYLCVSHLIGWTAFLSNMLFFTDFMGQIVYHGDPYAPHNSTSFLIYERG	

(G)

RGS7BP

M. rufoniger	TRTKGCEMARQAHQKLAAISGPEGGEIHPEICRLYIQLQCCLEMYTTEML	140
M. davidii	------------------------------------MISCLPPLYQHPLP	
M. brandtii	TRTKGCEMARQAHQKLAAISGPEGGEIHPEICRLYIQLQCCLEMYTTEML	
M. lucifugus	TRTKGCEMARQAHQKLAAISGPEGGEIHPEICRLYIQLQCCLEMYTTEML	
E. fuscus	TRTKGCEMARQAHQKLAAISGPEGGEIHPEICRLYIQLQCCLEMYTTEML	
P. alecto	TRTKGCEMARQAHQKLAAISGPEDGEIHPEICRLYIQLQCCLEMYTTEML	
P. vampyrus	TRTKGCEMARQAHQKLAAISGPEDGEIHPEICRLYIQLQCCLEMYTTEML	
R. aegyptiacus	TRTKGCEMARQAHQKLAAISGPEDGEIHPEICRLYIQLQCCLEMYTTEML	
B. taurus	TRTKGCEMARQAHQKLAAISGPEDGEIHPEICRLYIQLQCCLEMYTTEML	
E. caballus	TRTKGCEMARQAHQKLAAISGPEDGEIHPEICRLYIQLQCCLEMYATEML	
M. musculus	TRTKGCEMARQAHQKLAAISGPEDGEIHPEICRLYIQLQCCLEMYTTEML	
H. glaber	TRTKGCEMARQAHQKLAAISGPEDGEIHPEICRLYIQLQCCLEMYTTEML	
H. sapiens	TRTKGCEMARQAHQKLAAISGPEDGEIHPEICRLYIQLQCCLEMYTTEML	
M. domestica	TRTKGCEMARQAHQKLAAISGPEDGEIHPEICRLYIQLQCCLEMYTTEML	

(H)

RHO

M. rufoniger	PLNYILLNLAVANLFMVFGGFTTTLYTSMHGYFVFGATGCNLEGFFATLG	120
M. davidii	PLNYILLNLAVANLFMVFGGFTTTLYTSMHGYFVFGATGCNLEGFFATLG	
M. brandtii	PLNYILLNLAVANLFMVFGGFTTTLYTSMHGYFVFGATGCNLEGFFATLG	
M. lucifugus	PLNYILLNLAVANLFMVFGGFTTTLYTSMHGYFVFGATGCNLEGFFATLG	
E. fuscus	PLNYILLNLAVANLFMVFGGFTTTLYTSMHGYFVFGATGCNLEGFFATLG	
P. alecto	PLNYILLNLAVADLFMVFGGFTTTLYTSLHGYFVFGPTGCNLEGFFATLG	
P. vampyrus	------------------------------------MRQGSNQSLADCLA	
R. aegyptiacus	PLNYILLNLAVADLFMVFGGFTTTLYTSLHGYFVFGPTGCNLEGFFATLG	
B. taurus	PLNYILLNLAVADLFMVFGGFTTTLYTSLHGYFVFGPTGCNLEGFFATLG	
E. caballus	PLNYILLNLAVADLFMVFGGFTTTLYTSLHGYFVFGPTGCNVEGFFATLG	
M. musculus	PLNYILLNLAVADLFMVFGGFTTTLYTSLHGYFVFGPTGCNLEGFFATLG	
H. glaber	PLNYILLNLAVADLFMVICGFTTTLYTSMHGYFVFGATGCNMEGFFATLG	
H. sapiens	PLNYILLNLAVADLFMVLGGFTSTLYTSLHGYFVFGPTGCNLEGFFATLG	
M. domestica	PLNYILLNLIVADLFMVFGGFTMTLYTSLHGYFVFGPTGCNLEGFFATLG	

M. rufoniger	241	ATTQKAEKEVTRMVIIMVVAFLICWLPYASVAFYIFTHQG	280
M. davidii		ATTQKAEKEVTRMVIIMVVAFLICWLPYASVAFYIFTHQG	
M. brandtii		ATTQKAEKEVTRMVIIMVVAFLICWLPYASVAFYIFTHQG	
M. lucifugus		ATTQKAEKEVTRMVIIMVVAFLICWLPYASVAFYIFTHQG	
E. fuscus		ATTQKAEKEVTRMVIIMVVAFLICWLPYASVAFYIFTHQG	
P. alecto		ATTQKAEKEVTRMVIIMVIAFLICWLPYAGVAFYIFTHQG	
P. vampyrus		ATTQKAEKEVTRMVIIMVIAFLICWLPYAGVAFYIFTHQG	
R. aegyptiacus		ATTQKAEKEVTRMVIIMVIAFLICWLPYAGVAFYIFTHQG	
B. taurus		ATTQKAEKEVTRMVIIMVIAFLICWLPYAGVAFYIFTHQG	
E. caballus		ATTQKAEKEVTRMVIIMVIAFLICWVPYASVAFYIFTHQG	
M. musculus		ATTQKAEKEVTRMVIIMVIFFLICWLPYASVAFYIFTHQG	
H. glaber		ATTQKAEKEVTRMVIIMVIAFLICWVPYASVAMYIFTHQG	
H. sapiens		ATTQKAEKEVTRMVIIMVIAFLICWVPYASVAFYIFTHQG	
M. domestica		ATTQKAEKEVTRMVIIMVIAFLICWLPYAGVAFYIFTHQG	

(I)

OPN1SW

```
M. rufoniger      PWDGPQYHXAPVWAFHLQAAFMGFVFFAGTPLNATVLVATLRYKKLRQPL   70
M. davidii        PWDGPQYHIAPVWAFHLQAAFMGFVFFAGTPLNATVLVATLRYKKLRQPL
M. brandtii       PWDGPQYHIAPVWAFHLQAAFMGFVFFAGTPLNATVLVATLRYKKLRQPL
M. lucifugus      PWDGPQYHIAPVWAFHLQAAFMGFVFFAGTPLNATVLVATLRYKKLRQPL
E. fuscus         PWDGPQYHIAPVWAFHLQAAFMGFVFFAGTPLNATVLVATLRYKKLRQPL
P. alecto         PWDGPQHHIAPVWAFHLQAAFMGFVFFVGTPLNATVLVATLRYRKLRQPL
P. vampyrus       PWDGPQHHIAPVWAFHLQAAFMGFVFFVGTPLNATVLVATLRYRKLRQPL
R. aegyptiacus    PWDGPQYHVAPVWAFHLQAAFMGFVFFVGTPLNATVLVATLRYRKLRQLL
B. taurus         PWDGPQYHLAPVWAFHLQAVFMGFVFFVGTPLNATVLVATLRYRKLRQPL
E. caballus       PWDGPQYHIAPVWAFRLQAAFLGIVFLVGMPLNSLVLVATLRYKKLRQPL
M. musculus       PWDGPQYHLAPVWAFRLQAAFMGFVFFVGTPLNAIVLVATLHYKKLRQPL
H. glaber         PWDGSQYHIAPIWAFHLQAAFMGLVFFVGTPLNAIVLVATLQYKKLRQPL
H. sapiens        PWDGPQYHIAPVWAFYLQAAFMGTVFLIGFPLNAMVLVATLRYKKLRQPL
M. domestica      PWDGPQYHIAPAWAFHFQTVFMGFVFCAGTPLNAVVLVATLRYKKLRQPL
```

```
M. rufoniger      81  GFLFCIFSVFTVFIASCQGYFVFGRHVCALEAFLGSTAGL   120
M. davidii            GFLFCIFSVFTVFIASCQGYFVFGRHVCALEAFLGSTAGL
M. brandtii           GFLFCIFSVFTVFIASCHGYFVFGRHVCALEAFLGSTAGL
M. lucifugus          GFLFCIFSVFTVFIASCQGYFVFGRHVCALEAFLGSTAGL
E. fuscus             GFLFCIFSVFTVFIASCQGYFIFGRHVCALEAFLGSTAGL
P. alecto             GFLFCIFSVFTVFIASCQGYFVFGPHVCALEAFLGSTAGL
P. vampyrus           GFLFCIFSVFTVFIASCQGYFVFGRHVCALEAFLGSTAG-
R. aegyptiacus        GFLFCIFSVFTVFIISCQGYFVFGRHVCALEAFLGSAAGL
B. taurus             GFIYCIFSVFIVFITSCYGYFVFGRHVCALEAFLGCTAGL
E. caballus           GFLVCIFSVLIVFINSCHGYFVFGPHVCAFESFLGTVAGL
M. musculus           GFLFCIFSVFTVFIASCHGYFLFGRHVCALEAFLGSVAGL
H. glaber             GFLFCIFSVFTVFIASCHGYFFFGRHVCALEAFLGSVAGL
H. sapiens            GFLLCIFSVFPVFVASCNGYFVFGRHVCALEGFLGTVAGL
M. domestica          GFIFCLFAVFTVFISSSQGYFIFGRHVCAMEAFLGSVAGL
```

(J)

CNGB3

M. rufoniger	FFEFNHRLESIMNRAYIYRVARTAGCLLFALHVNACIYYWASDHQGIGTT	430
M. davidii	FFEFNHHLESIMNRAYIYRVARTAGCLLFALHVNACIYYWASDHQGFGST	
M. brandtii	FFEFNHCLESIMNRAYIYRVARTAGCLLFALHVNACIYYWASDHQGIGTT	
M. lucifugus	FFEFNHRLESIMNRAYIYRVARTAGCLLFALHVNACIYYWASNHQGIGTT	
E. fuscus	FFEFNHRLESIMDKAYIYRVARTAGCLLFALHVNACIYYWASDHQGIGTT	
P. alecto	FFEFNHRLESAMDKAYVYRVFRTTGYLLFTLHINACIYYWASAYEGLGTT	
P. vampyrus	FFEFNHRLESAMDKAYVYRVFRTTGYLLFTLHINACIYYWASAYEGLGTT	
R. aegyptiacus	FFEFNHRLESVMDKAYIYRVLRTTGYLLFTLHINACIYYWASAYEGLGTT	
B. taurus	--	
E. caballus	FFEFNHHLESIMDKAYIYRVIRTTGYLLFTLHINACIYYWASNYEGIGTT	
M. musculus	FFEFNHHLESIMDKAYVYRVIRTTGYLLFLLHINACVYYWASDYEGIGST	
H. glaber	FFEFNHHLESIMDKVYIYRVIRTTGYLLFILHINACIYYWASNYEGIGTT	
H. sapiens	FFEFNHHLESIMDKAYIYRVIRTTGYLLFILHINACVYYWASNYEGIGTT	
M. domestica	FFEFNDRLESRMDNAYIYRVIRTTGYLLFVLHVNACIYSLASDYEGIGST	
