# Supplementary material for: Myotis rufoniger genome sequence and analyses: M. rufoniger’s genomic feature and the decreasing effective population size of Myotis bats
Source: PLoS One. 2017 Jul 5;12(7):e0180418. doi: 10.1371/journal.pone.0180418 (PMC5498047; doi:10.1371/journal.pone.0180418)
Supplement: S4 Fig — Myotis bats’ uAACs within DCT, SLC45A2, TYRP1, and OCA2 genes are highlighted (yellow if PROVEAN score of variant ≤ -2.5, unless green). The domain region of human sequences are shaded in gray; (A) Alignment of DCT encoded peptide sequences; (B) Alignment of SLC45A2 encoded peptide sequences; (C) Alignment of TYRP1 encoded peptide sequences; (D) Alignment of OCA2 encoded peptide sequences. (PDF) [file pone.0180418.s006.pdf]

(A)

DCT

```
                  1                                                    50
M. rufoniger     --MLKSSSGR QEEX-----S IGGESKFPDC KAMRSLWWGL LLSWLGCGML
M. davidii       --MLKSSSGR QEES-----S IGGERKFPDC KAMRSLWWGL LLSWLGCGML
M. brandtii      --MLKSSSGR QEES-----S KGGESKFPDC KAMRSLWWGL LLSWLGCGML
M. lucifugus     --MLKSSSGR QEES-----S IGGESKFPDC KAMRSLWWGL LLSWLGCGML
E. fuscus        ---------- ---------- ---------- --MRSLWWGL LLSWLGCGML
P. alecto        ---------- ---------- ---------- --MSPLRWGL LLSCLSCGIL
P. vampyrus      ---------- ---------- ---------- --MSPLRWGL LLSCLSCGIL
R. aegyptiacus   ---------- ---------- ---------- --MGPLLWGL LLSCLGCGIL
B. taurus        ---------- ---------- ---------- --MSPLGWGL LLGCLGCALP
E. caballus      MNAQEPKSGR QGERQDRSKE WKVQESSPGY KAMSSLRWGL LLSCLGCGLL
M. musculus      ---------- ---------- ---------- --MGLVGWGL LLGCLGCGIL
H. glaber        ---------- ---------- ---------- --MRPLQWGL LLGCLGSCLW
H. sapiens       ---------- ---------- ---------- --MSPLWWGF LLSCLGCKIL
M. domestica     ---------- ---------- -------MIK QQQAGLPWWL TLLCLGSCLL

                 51                                                   100
M. rufoniger     PGARAQFPRV CMTVASLVAK ECCPPLGVEP TNVCGSQEGR GQCTEVQADT
M. davidii       PGARAQFPRV CMTVASLVAK ECCPPLGVEP ANVCGSQEGR GQCTEVQADT
M. brandtii      PGAGAQFPRV CMTVASLVAK ECCPPLGVEP ANICGSQEGR GQCTEVQADT
M. lucifugus     PGARAQFPRV CMTVASLVAK ECCPPLGVEP ANVCGSQEGR GQCTEVQADT
E. fuscus        PGAWAQFPRV CMTVASLVAK ECCPPLGVEP ANVCGSQEGR GQCTEVQADT
P. alecto        PGVWAQFPRV CMTMDNLVSK ECCPPLGAEP GNVCGSQAGR GQCTEVQTDT
P. vampyrus      PGVWAQFPRV CMTMDNLVSK ECCPPLGVEP GNVCGSQTGR GQCTEVQTDT
R. aegyptiacus   PGVWAQFPRV CMTVDNLVSK ECCPPLGAEP DNVCGSRAGR GQCTEVQTDT
B. taurus        SGARAQFPRV CMTVGSLQAK ECCPPLGADP ANVCGSREGR GQCAEVQTDT
E. caballus      PGAWAQFPRV CMTVDSLLSK ECCPPLGMEP ANVCGSQEGR GQCTEVETDT
```

M. musculus	LRARAQFPRV CMTLDGVLNK ECCPPLGPEA TNICGFLEGR GQCAEVQTDT
H. glaber	PGAWAQFPRV CMTLDSLRGK ECCPPLGVEP TNVCGSQDGR GQCLEVRADS
H. sapiens	PGAQGQFPRV CMTVDSLVNK ECCPRLGAES ANVCGSQQGR GQCTEVRADT
M. domestica	PMTWAQFPRT CMTLESVVSK ECCPALGVDP ANRCGMLEGR GQCAEVQTDA

Topological domain: Lumenal, melanosome

	101 150
M. rufoniger	RPWSGPYVLR NQDDREWWPR KFFNRTCNCT GNFAGFNCGD CKFGWTGPNC
M. davidii	RPWSGPYVLR NQDDRERWPR KFFNRTCNCT GNFAGFNCGD CKFGWTGPNC
M. brandtii	RPWSGPYVLR NQDDRERWPR KFFNRTCNCT GNFAGFNCGD CKFGWTGPNC
M. lucifugus	RPWSGPYVLK NQDDRERWPR KFFNRTCNCT GNFAGFNCGD CKFGWTGPNC
E. fuscus	RPWSGPYVLR NQDDRERWPR KFFNRTCKCT GNFSGFNCGD CKFGWTGPNC
P. alecto	RPWGGPYVLR NQDDRERWPR KFFNRTCKCT GNFAGYNCGG CKFGWIGLNC
P. vampyrus	RPWGGPYVLR NQDDRERWPR KFFNRTCKCT GNFAGYNCGG CKFGWIGLNC
R. aegyptiacus	RPWGGPYVLR NQDDRERWPR KFFNRTCKCT GNFAGYNCGG CKFGWIGLNC
B. taurus	RPWSGPYVLR NQDDRERWPR KFFDRTCRCT GNFAGYNCGN CRFGWTGPKC
E. caballus	RPWSGPYILR NQDDRERWPR KFFDWTCKCT GNFAGYNCGD CKFGWTGPNC
M. musculus	RPWSGPYILR NQDDREQWPR KFFNRTCKCT GNFAGYNCGG CKFGWTGPDC
H. glaber	RPWSGPYVLR NRDDREQWPR KFFHRTCKCT GNFAGYHCGD CRFGWTGPNC
H. sapiens	RPWSGPYILR NQDDRELWPR KFFHRTCKCT GNFAGYNCGD CKFGWTGPNC
M. domestica	RPWSGPYVLR NQDDREQWPR KFFNRTCRCT DNFAGYNCGD CKFGWTGPTC

Topological domain: Lumenal, melanosome

	151 200
M. rufoniger	DQKKPPVIRK NIHFLTPQER EQFLGALDLA KNTTHPDYVI TTQHWLGLLG
M. davidii	DQKKPPVIRK NIHFLTPQER EQFLGALDLA KNTTHPDYVI TTQHWLGLLG
M. brandtii	DQKKPPVIRK NIHFLTPQER EQFLGALDLA KNTTHPDYVI TTQHWLGLLG
M. lucifugus	DQKKPPVIRK NIHFLTPQER EQFLGALDLA KNTTHPDYVI TTQHWLGLLG
E. fuscus	DQKKPPVVRK NIHFLTPQER EQFLGALDLA KNTTHPDYVI TTQHWLGLLG
P. alecto	DRKKPPVVRQ NIHSLTPQER EQFLGALDLA KKTIHPDYVI TTQHWLGLLG
P. vampyrus	DRKKPPVVRQ NIHSLTPQER EQFLGALDLA KKTIHPDYVI TTQHWLGLLG
R. aegyptiacus	DRKKPPVVRQ NIHSLTPQER EQFLGALDLA KKTIHPDYVI TTQHWLGLLG
B. taurus	DQKKPLVVRR DVHSLTPQER EQFLDALDLA KYTPHPDYVI TTQHWLGLLG

E. caballus	DRKKAPVVRQ NIHSLTPQER EQFLGALDLA KNTTHPDYVI TTQHWLGLLG
M. musculus	NRKKPAILRR NIHSLTAQER EQFLGALDLA KKSIHPDYVI TTQHWLGLLG
H. glaber	DQKKPPVVRQ NIHSLTPQER EQFLGALDLA KKTTHPDYVI TTQHWLGLLG
H. sapiens	ERKKPPVIRQ NIHSLSPQER EQFLGALDLA KKRVHPDYVI TTQHWLGLLG
M. domestica	NQKKPPVVRK NIHSLSPRER EQFLDALDLA KNTIHPDYVI TTQHWLGLLG

Topological domain: Lumenal, melanosome

	201 ... 250
M. rufoniger	PNGTQPQIAN CSIYDFFVWL HYYSVRDTLL GPGRPYKAID FSHQGPAFVT
M. davidii	PNGTQPQIAN CSIYDFFVWL HYYSVRDTLL GPGRPYKAID FSHQGPAFVT
M. brandtii	PNGTQPQIAN CSIYDFFVWL HYYSVRDTLL GPGRPYKAID FSHQGPAFVT
M. lucifugus	PNGTQPQIAN CSIYDFFVWL HYYSVRDTLL GPGRPYKAID FSHQGPAFVT
E. fuscus	PNGTQPQIAN CSIYDFFVWL HYYSVRDTLL GPGRPYKAID FSHQGPAFVT
P. alecto	PNRTQPQIAN CSIYDFFVWL HYYSVRDTLL GPGRPYKAID FSHQGPAFVT
P. vampyrus	PNRTQPQIAN CSIYDFFVWL HYYSVRDTLL GPGRPYKAID FSHQGPAFVT
R. aegyptiacus	PNGTQPQIAN CSIYDFFVWL HYYSVRDTLL GPGRPYKAID FSHQGPAFVT
B. taurus	PNGTQPQIAN CSIYDFFVWL HYYSVRDTLL GPGRPYKAID FSHQGPAFVT
E. caballus	PNGTQPQIAN CSIYDFFVWL HYYSVRDTLL GPGRPYKAID FSHQGPAFVT
M. musculus	PNGTQPQIAN CSVYDFFVWL HYYSVRDTLL GPGRPYKAID FSHQGPAFVT
H. glaber	PQGTQPQIAN CSIYDFFVWL HYYSVRDTLL GPGRPYKAVD FSHQGPAFVT
H. sapiens	PNGTQPQFAN CSVYDFFVWL HYYSVRDTLL GPGRPYRAID FSHQGPAFVT
M. domestica	PNGTQPQIAN CSIYNYFVWL HYYSVRDTLL GPGRPFKAID FSHQGPAFLT

Topological domain: Lumenal, melanosome

	251 ... 300
M. rufoniger	WHRYHLLWLE RDLQRLIGNE SFALPYWNFA TGRNECDVCT DQLLGAARQD
M. davidii	WHRYHLLCLE RDLQRLIGNE SFALPYWNFA TGRNECDVCT DQLLGAARQD
M. brandtii	WHRYHLLWLE RDLQRLIGNE SFALPYWNFA TGRNECDVCT DQLLGAARQD
M. lucifugus	WHRYHLLWLE RDLQRLIGNE SFALPYWNFA TGRNECDVCT DQLLGAARQD
E. fuscus	WHRYHLLWLE RDLQRLIGNE SFALPYWNFA TGRNECDVCT DQLFGAARQD
P. alecto	WHRYHLLWLE RDLQRLIGNE SFALPYWNFA TGRNECDVCT DQLFGAARQD
P. vampyrus	WHRYHLLWLE RDLQRLIGNE SFALPYWNFA TGRNECDVCT DQLFGAARQD
R. aegyptiacus	WHRYHLLWLE RDLQRLIGNE SFALPYWNFA TGRNECDVCT DQLFGAARQD

B. taurus	WHRYHLLWLE RDLQRLTGNE SFALPYWNFA TGRNECDVCT DQLLGAARQD
E. caballus	WHRYHLLWLE RDLQRLTGNE SFALPYWNFA TGRNECDVCT DQLLGAARQD
M. musculus	WHRYHLLWLE RELQRLTGNE SFALPYWNFA TGKNECDVCT DELLGAARQD
H. glaber	WHRYHLLWLE RDLQRLTGNE SFALPYWNFA TGRNECDVCT DQLLGAARQD
H. sapiens	WHRYHLLCLE RDLQRLIGNE SFALPYWNFA TGRNECDVCT DQLFGAARPD
M. domestica	WHRYHLLLLE RDLQRLTGNE SFALPYWNFA TGKNECDVCT DQLFGASRLE

Topological domain: Lumenal, melanosome

	301 350
M. rufoniger	DPTLISQNSR FSSWEVVCDS LDDYNHRVTL CNGTYEGLLR RNQAGRNGEK
M. davidii	DPTLISQNSR FSSWEIVCDS LDDYNRRVTL CNGTYEGLLR RNQAGRNGEK
M. brandtii	DPTLISQNSR FSSWEIVCDS LDDYNRRVTL CNGTYEGLLR RNQAGRNGEK
M. lucifugus	DPTLISQNSR FSSWEIVCDS LDDYNRRVTL CNGTYEGLLR RNQAGRNGEK
E. fuscus	DPTLISQNSR FSSWEIVCDS LDDYNRRVTL CNGTYEGLLR RNQAGRNGEK
P. alecto	DPTLISQNSR FSSWEIVCNS LDDYNNRVTL CNGTYEGLLR RNRVGRNSEK
P. vampyrus	DPTLISQNSR FSSWEIVCNS LDDYNNRVTL CNGTYEGLLR RNRVGRNSEK
R. aegyptiacus	DPTLISQNSR FSSWEIVCDS LDDYNRRVTL CNGTYEGLLR RNRVGRNSEK
B. taurus	DPTLISQNSR FSSWEIVCDS LNDYNRRVTL CNGTYEGLLK RNQMGRNSEK
E. caballus	DPMLISQNSR FSSWEIVCDS LDDYNRRVTL CNGTYEGLLR RNQVGRNSEK
M. musculus	DPTLISRNSR FSTWEIVCDS LDDYNRRVTL CNGTYEGLLR RNKVGRNNEK
H. glaber	DPTLISQNSR FSSWEIVCDS LDDYNQRVTL CNGTYEGLLR RNQMGRNREK
H. sapiens	DPTLISRNSR FSSWETVCDS LDDYNHLVTL CNGTYEGLLR RNQMGRNSMK
M. domestica	DSALISQNSR FSRWEIVCNS LDDYNRQVTL CNGTNEGLLR RNQLGRTRVQ

Topological domain: Lumenal, melanosome

	351 400
M. rufoniger	LPSLKDIEDC LSLKQFDNPP FFQNSTFSFR NALEGFDKAD GTLDSQVMSL
M. davidii	LPSLKDIEDC LSLKQFDNPP FFQNSTFSFR NALEGFDKAD GTLDSQVMSL
M. brandtii	LPSLKDIEGC LSLKQFDNPP FFQNSTFSFR NALEGFDKAD GTLDSQVMSL
M. lucifugus	LPSLKDIEGC LSLKQFDNPP FFQNSTFSFR NALEGFDKAD GTLDSQVMSL
E. fuscus	LPSLKDIEDC LSLKKFDNPP FFQNSTFSFR NALEGFDKAD GTLDSQVMSL
P. alecto	LPTLKEIQDC LSLKKFDNPP FFQNSTFSFR NALEGFDKAD GTLDSQAMSL
P. vampyrus	LPTLKEIQDC LSLKKFDNPP FFQNSTFSFR NALEGFDKAD GTLDSQAMSL

R. aegyptiacus	LPNLKDIQDC LSLTKFDNPP FFQNSTFSFR NALEGFDKAD GTLDSQVMSL
B. taurus	LPTLKDIQNC LSLKKFDSPP FFQNSTLSFR NALEGFGKAD GTLDSQVMNF
E. caballus	LPTLHDIQDC LSLKKFDNPP FFQNSTFSFR NALEGFDKAD GTLDSQVLSL
M. musculus	LPTLKNVQDC LSLQKFDSPP FFQNSTFSFR NALEGFDKAD GTLDSQVMNL
H. glaber	LPTLKDVQDC LSLQKFDNPP FFQNSTFSFR NALEGFDKAD GTLGSQVASL
H. sapiens	LPTLKDIRDC LSLQKFDNPP FFQNSTFSFR NALEGFDKAD GTLDSQVMSL
M. domestica	LPTIEDVQAC LSLEKFDNPP FFRNSSFSFR NALEGFDKAD GTFDSPVMSL

Topological domain: Lumenal, melanosome

	401 450
M. rufoniger	HNLVHSFLNG TNALPHSAAN DPIFVV---- ---------- ----------
M. davidii	HNLVHSFLNG TNALPHSAAN DPIFVV---- ---------- ----------
M. brandtii	HNLVHSFLNG TNALPHSAAN DPIFVV---- ---------- ----------
M. lucifugus	HNLVHSFLNG TNALPHSAAN DPIFVV---- ---------- ----------
E. fuscus	HNLVHSFLNG TNALPHSAAN DPIFVV---- ---------- ----------
P. alecto	HNLVHSFLNG TSALPHSAAN DPIFVV---- ---------- ----------
P. vampyrus	HNLVHSFLNG TSALPHSAAN DPIFVV---- ---------- ----------
R. aegyptiacus	HNLVHSFLNG TNALPHSAAN DPIFVV---- ---------- ----------
B. taurus	HNLVHSFLNG TSALPHSAAN DPVFVV---- ---------- ----------
E. caballus	HNLVHSFLNG TSALPHSAAN DPIFVV---- ---------- ----------
M. musculus	HNLAHSFLNG TNALPHSAAN DPVFVV---- ---------- ----------
H. glaber	HNLVHSFLNG TSALPHSAAN DPVFVV---- ---------- ----------
H. sapiens	HNLVHSFLNG TNALPHSAAN DPIFVVISNR LLYNATTNIL EHVRKEKATK
M. domestica	HNLVHAFLNG TSALPHSAAN DPIFVV---- ---------- ----------

Topological domain: Lumenal, melanosome

	451 500
M. rufoniger	---------- ---------L HAFTDAIFDE WMRRFNPSXD AWPQELAPIG
M. davidii	---------- ---------L HAFTDAIFDE WMRRFNPSAD AWPQELAPIG
M. brandtii	---------- ---------L HAFTDAIFDE WMRRFNPSAD AWPQELAPIG
M. lucifugus	---------- ---------L HAFTDAIFDE WMRRFNPSAD AWPQELAPIG
E. fuscus	---------- ---------L HAFTDAIFDE WMRRFNPSAD AWPQELAPIG
P. alecto	---------- ---------L HSFTDAIFDE WMKRFNPSAN AWPQELAPIG

P. vampyrus ---------- ---------L HSFTDAIFDE WMKRFNPSAN AWPQELAPIG

R. aegyptiacus ---------- ---------L HCFTDAIFDE WMKRFNPSVD AWPQELAPIG

B. taurus ---------- ---------L HSFTDAIFDE WMKRFNPPVD AWPRELAPIG

E. caballus ---------- ---------L HSFTDAIFDE WMRRFNPPAE AWPQELAPIG

M. musculus ---------- ---------L HSFTDAIFDE WLKRNNPSTD AWPQELAPIG

H. glaber ---------- ---------L HSFTDSIFEE WMKRYKPAAD AWPQELAPIG

H. sapiens ELPSLHVLVT MSLGGSTQVL HSFTDAIFDE WMKRFNPPAD AWPQELAPIG

M. domestica ---------- ---------L HSFTDAIFDE WMKRFNPPVN AWPPELAPIG

Topological domain: Lumenal, melanosome

501 550

M. rufoniger HNRMHNMVPF FPSVTNQELF LTADQLGYSY AIDLPV--EE T--PSWSVTL

M. davidii HNRMYNMVPF FPSVTNQELF LTADQLGYSY AIDLPV--EE T--PSWSVTL

M. brandtii HNRMYNMVPF FPSVTNQELF LTADQLGYSY AIDLPV--EE T--PSWSVTL

M. lucifugus HNRMYNMVPF FPSVTNQELF LTADQLGYSY AIDLPV--EE T--PSWSVTL

E. fuscus HNRMYNMVPF FPSVTNQECF LTTDQLGYSY AIDLPVSVEE T--PSWSTTL

P. alecto HNRMYNMVPF FPPVTNEEFF LTADQLGYSY VIDLPVSVEE T--PSWNTTL

P. vampyrus HNRMYNMVPF FPPVTNEEFF LTADQLGYSY VIDLPVSVEE T--PSWNTTL

R. aegyptiacus HNRMYNMVPF FPPVTNEEFF LTADQLGYSY VIDLPV--EE T--PSWNTTL

B. taurus HNRMYNMVPF FPPVTNEELF LTADQLGYSY AIDLPV--EE T--PDWTTVL

E. caballus HNRMYNMVPF FPPVTNEELF LTADQLGYSY AIDLPV--EQ T--PGWTTTL

M. musculus HNRMYNMVPF FPPVTNEELF LTAEQLGYNY AVDLSE--EE A--PVWSTTL

H. glaber HNRMYNMVPF FPPVTNEELF LTADQLGYSY AIDLPVSVEE T--PSWITTL

H. sapiens HNRMYNMVPF FPPVTNEELF LTSDQLGYSY AIDLPVSVEE T--PGWPTTL

M. domestica HNRMYNMVPF FPPVTNEEFF LTADQLGYDY SINLSASSSG KATQDQTLIL

Topological domain: Lumenal, melanosome

551 600

M. rufoniger PVVMGMLVAL VGLFVLLAFL QYRRLHKGYA PLMETQFN-K RYTEEA----

M. davidii PVVLGMLVAL VGLFVLLAFL QYRRLRKGYA PLMETHLN-K RYTEEA----

M. brandtii PVVMGMLVAL VGLFVLLAFL QYRRLRKGYA PLMETHWN-K RYTEEA----

M. lucifugus PVVMGMLVAL VGLFVLLAFL QYRRLRKGYA PLMETHLN-K RYTEEA----

E. fuscus PVVMGMLVVL VGLFVLLAFL QYRRLRKGYA PLMETHLNNK RYTEEA----

P. alecto	SVVVGILVAL IGLLVLLAFL QYRRLRKGYA PLMETHLSNK RYMEEA----
P. vampyrus	SLVMGILVAL IGLLVLLAFL QYQRLRKGYA PLMETHLSNK RYMEEA----
R. aegyptiacus	SVVMGMLVAL IGLLVLLAFL QYRRLRKGYA PLMETHLSNK RYTEEA----
B. taurus	SVVTGMLVVL VVLFALLLFL QYRRLRKGYT PLVETQLSNK RYTEEA----
E. caballus	SVVMGMLVAL VGLFALLVFL QYRRLRKGYT PLMETHLSK- RYTEEA----
M. musculus	SVVIGILGAF VLLLGLLAFL QYRRLRKGYA PLMETGLSSK RYTEEA----
H. glaber	SVVMGILVAL VGLFMLLIFL QYRRLRKGYT PLMETHLSNK RYTKEAERVQ
H. sapiens	LVVMGTLVAL VGLFVLLAFL QYRRLRKGYT PLMETHLSSK RYTEEA----
M. domestica	SVIGAGLVAL VALSLLLVLL QYRRQRKGFA PLMETRFSNR KYTEDA----

Topological domain: Cytoplasmic

	601
M. rufoniger	-
M. davidii	-
M. brandtii	-
M. lucifugus	-
E. fuscus	-
P. alecto	-
P. vampyrus	-
R. aegyptiacus	-
B. taurus	-
E. caballus	-
M. musculus	-
H. glaber	A
H. sapiens	-
M. domestica	-

(B)

SLC45A2

	1				50
M. rufoniger	MKESEPFPQP	NSLPGSKS--	----KXAHKS	EGPRRRWRAC	RQVPHPAAMG
M. davidii	----------	----------	----------	----------	--------MG
M. brandtii	MKESEPFPQP	NPLPGSKS--	----KPAHKS	EGPRRRWRAG	RQVPHPVAMG
M. lucifugus	----------	----------	----------	----------	--------MG
E. fuscus	----------	----------	----------	----------	--------MG
P. alecto	----------	----------	----------	----------	----------
P. vampyrus	----------	----------	----------	----------	--------MG
R. aegyptiacus	----------	----------	----------	----------	--------MG
B. taurus	MKEFEPRPQP	GRLCGPCSQA	PCQIHLGART	PGPRRRWWAC	RQVPRPVAMG
E. caballus	MKEFEPLPQP	DHLCWLLSQA	LCQIQLEAQT	RGPPGWRWAC	RQVPHPVAMG
M. musculus	----------	----------	----------	----------	--------MS
H. glaber	----------	----------	----------	----------	--------MG
H. sapiens	----------	----------	----------	----------	--------MG
M. domestica	----------	----------	----------	----------	--------MG

	51				100
M. rufoniger	GNSGQPGTHX	YTSLAEDGPL	AAGEQPRRPT	GRLIMHSMAM	FGREFCYAVE
M. davidii	GNSGQPGTHT	YRSLAEDGPL	AAGEPPRRPT	GRLIMHSMAM	FGREFCYAVE
M. brandtii	GNSGQPGTHT	YTSLTEDGPL	AAGEQPRRPT	GRLIMHSMAM	FGREFCYAVE
M. lucifugus	GNSGQPGTHT	YTSLEEDGPL	AAGEQPRRPT	GRLIMHSMAM	FGREFCYAVE
E. fuscus	GNTGQPGMHS	YTSLAEDGPW	AAGEQPKRPT	GRLIMHSMAM	FGREFCYAVE
P. alecto	----------	----------	----------	-------MAM	FGREFCYAVE
P. vampyrus	GNSGQLSIHT	YTSLGEDGPF	GFVEPPKRLT	GRLIMHSMAM	FGREFCYAVE
R. aegyptiacus	GNSGQPSIHT	YTSLAEDGPF	GFVEAPKRPT	GRLIMHSMAM	FGREFCYAVE
B. taurus	DKSGQSPLRT	YKSLDEEGLF	GSAELPKRPT	GSLVMHSMAM	FGREFCYAVE
E. caballus	GNSGQPGVPT	YKSLAEDGPF	GSVELPKRST	GRLVMHSMAM	FGREFCYAVE

```
M. musculus     GSNGPTDTHT YQSLAEDCPF GSVEQPKRST GRLVMHSMAM FGREFCYAVE
H. glaber       SNGGQAGTHT YQSLTEDALF GSMEPPRRPT GRLVMHSLAM FGREFCYAVE
H. sapiens      SNSGQAGRHI YKSLADDGPF DSVEPPKRPT SRLIMHSMAM FGREFCYAVE
M. domestica    VSGTQTGNPK YTSLATAGSL GHAEPPRRST GRLVMHSMAM LGREFCYAVE

                101                                              150
M. rufoniger    AAYVTPVLLS VGLPKSLYSV VWLLSPILGF LLQPVVGSAS DYCRAGWGRR
M. davidii      AAYVTPVLLS VGLPKSLYSV VWLLSPILGF LLQPVVGSAS DYCRAGWGRR
M. brandtii     AAYVTPVLLS VGLPKSLYSV VWLLSPVLGF LLQPVVGSAS DYCRAGWGRR
M. lucifugus    AAYVTPVLLS VGLPKSLYSV VWLLSPVLGF LLQPVVGSAS DYCRAGWGRR
E. fuscus       AAYVTPVLLS VGLPKSLYSV VWLLSPVLGF LLQPVVGSAS DYCRAGWGRR
P. alecto       AAYVTPVLLS VGLPRSLYSM VWLLSPVLGF LLQLVVGSAS DHCHATWGRR
P. vampyrus     ASYVTPVLLS VGLPKSLYSM VWLLSPVLGF LLQLVVGSAS DHCHATWGRR
R. aegyptiacus  AAYVTPVLLS VGLPRRLYSV VWLLSPILGF LLQPVVGSAS DHCHAMWGRR
B. taurus       AAYVTPVLLS VGLPKSLYSM VWLLSPILGF LLQPVVGSAS DHCRARWGRR
E. caballus     AAYVTPVLLS VGLPKRLYSV VWLLSPVLGF LLQPVVGSAS DHCRARWGRR
M. musculus     AAYVTPVLLS VGLPKSLYSM VWLLSPILGF LLQPVVGSAS DHCRARWGRR
H. glaber       AAYVTPVLLS VGLPESLYSV VWLLSPILGF LLQPVVGSAS DHCQSRWGRR
H. sapiens      AAYVTPVLLS VGLPSSLYSI VWFLSPILGF LLQPVVGSAS DHCRSRWGRR
M. domestica    AAYVTPVLLS VGLPQSLYSV VWLISPILGF MLQPIVGSAS DHCRSRWGKR

                151                                              200
M. rufoniger    RPYILALGVM MLLGMALYLN GDTVVSALTV DPRRKL-VWA ITITMMGVVV
M. davidii      RPYILALGVM MLLGMALYLN GDTVVSALIA DPRRKL-IWA ITITMMGVVL
M. brandtii     RPYILALGVM MLLGMALYLN GDTVVSALIA DPRRKL-IWA ITITMMGVVL
M. lucifugus    RPYILALGIM MLLGMALYLN GDTVVSALIA DPRRKL-IWA ITITMMGVVL
E. fuscus       RPYILALGVM MLLGMALYLN GDTVVSVLIA DPRRKL-IWA ITITMMGVVL
P. alecto       RPYILTLAVM MLLGMGFYLN GDAIVSALIA DPRRRL-IWT ITITMLGVVL
P. vampyrus     RPYILTLAVM MLLGMAFYLN GDAIVSALIA DPRRRL-IWT ITITMIGVVL
R. aegyptiacus  RPYILTLAVM MLLGMALYLN GDTVVSALIA DPRRRL-IWA ITITMIGVVL
B. taurus       RPYILTLGLM MLLGMAMYLN GDAIISALIA DPRRKP-IWA ISITMIGVVL
```

E. caballus   RPYILALSVI MLLGMALYLN GDAVISALIA DRRKKL-TWA ITITMIGVVL

M. musculus   RPYILTLAIM MLLGMALYLN GDAVVSALVA NPRQKL-IWA ISITMVGVVL

H. glaber   RPYILTLGIM MLLGMALYLN GDAVVSALIL NPRRKL-VWA ISVTMIGVVL

H. sapiens   RPYILTLGVM MLVGMALYLN GATVVAALIA NPRRKL-VWA ISVTMIGVVL

M. domestica   RPYILTLGIL MLLGMALYLN GDAVVSAVFT KPSKKLTMWA VSITMIGVVL

Transmembrane: Helical

201 250

M. rufoniger   FDFAADFVDG PIKAYLFDVC SHQDKERGLH YHALFTGFGG ALGYLLGAID

M. davidii   FDFAADFIDG PIKAYLFDVC SHQDKERGLH YHALFTGFGG ALGYLLGAID

M. brandtii   FDFAADFIDG PIKAYLFDVC SHRDKERGLH YHALFTGFGG ALGYLLGAID

M. lucifugus   FDFAADFIDG PIKAYLFDVC SHQDKERGLH YHALFTGFGG ALGYLLGAID

E. fuscus   FDFAADFIDG PIKAYLFDVC SHQDKERGLH YHALFTGFGG ALGYLLGAID

P. alecto   FDFAADFIDG PIKAYLFDVC SHQDKERGLF YHALFTGFGG ALGYLLGAID

P. vampyrus   FDFAADFIDG PIKAYLFDVC SHQDKERGLY YHALFTGFGG ALGYLLGAID

R. aegyptiacus   FDFAADFIDG PIKAYLFDVC SHQDKERGLH YHALFTGFGG ALGYLLGAID

B. taurus   FDFAADFIDG PIKAYLFDVC THRDKERGLH FHALFTGLGG ALGYLLGAID

E. caballus   FDFAADFIDG PIKAYLFDVC SHQDKERGLH HHALFTGLGG ALGYILGAID

M. musculus   FDFSADFIDG PIKAYLFDVC SHQDKEKGLH YHALFTGFGG ALGYILGAID

H. glaber   FDFAADFIDG PIKAYLFDVC SHRDKERGLH YHALFTGVGG ALGYLLGAID

H. sapiens   FDFAADFIDG PIKAYLFDVC SHQDKEKGLH YHALFTGFGG ALGYLLGAID

M. domestica   FDFAADFIDG PIKAYLFDVC SYQDKEKGLH YHALFTGIGG ALGYVLGAID

Transmembrane: Helical

251 300

M. rufoniger   WAHLELGRVL GTEFQVMFFF SALVLTLCFI IHLCSIPEAP LRDTAKDSLL

M. davidii   WAHLKLGRVL GTEFQVMFFF SALVLTLCFI IHLCSIPEAP LRDTAKDSLL

M. brandtii   WAHLKLGRVL GTEFQVMFFF SALVLTLCFI IHLCSIPEAP LRDTAKDSLL

M. lucifugus   WAHLKLGRVL GTEFQVMFFF SALVLTLCFI IHLCSIPEAP LRDTAKDSLL

E. fuscus   WAHLKLGRVL GTEFQVMFFF SALVLTLCFI IHLCSIPEAP LRDAARDVLP

P. alecto   WAHLKLGRVL GTEFQVMFFF SASVLILCFI IHLCSIPEAP LRDVTKDIPP

P. vampyrus   WAHLKLGRVL GTEFQVMFFF SALVLILCFI IHLCSIPEAP LRDVTKDIPP

R. aegyptiacus   WAHLKLGRVL GTEFQVMFFF SALVLTLCFI IHLCSIPEAP LRDVTKDIPP

```
B. taurus        WAHLELGRLL GTEFQVMFFF SSLVLTLCFI IHLCSIPEAP LRDVAKDIPP
E. caballus      WAHLKLGRML GTEFQVMFFF SALMLTLCVV IHLCSIPEAP LRDVAKDIPP
M. musculus      WVHLDLGRLL GTEFQVMFFF SALVLILCFI THLCSIPEAP LRDAATDPPS
H. glaber        WAHLELGRVL GTEFQVMFFF SALMLTLCFI IHLCSIPEAP LRDVAKDISP
H. sapiens       WAHLELGRLL GTEFQVMFFF SALVLTLCFT VHLCSISEAP LTEVAKGIPP
M. domestica     WGHLELGRLL GTEFQVMFFF SVLVFTICLI IHLCSIPEAP LCDNPEDSTL

                 301                                                350
M. rufoniger     QQAPQDPPLP SDKTYEYGSI EKVKNGYVHS ELT-----VQ GEKNKNPAEQ
M. davidii       QQAPQDPPLS SDKTYEYGSI EKVKNGYVHS ELT-----VQ GEKTKNPAKQ
M. brandtii      QQAPQDPPLS SDKTYEYGSI EKVKNGYVHS ELT-----VQ GEKTKNPAEQ
M. lucifugus     QQAPQDPPLS SDKAYEYGSI EKVKNGYVHS ELT-----VQ GEKTKNPAEQ
E. fuscus        QQAPQGAPLS ADKTYEYGSI EKVKNGDVHS ELT-----MQ GEKDTNPAEQ
P. alecto        QQAPQNPPLS SDRMYEYGSI EKVKNDSVNP ELV-----ML GEKTKNPAAQ
P. vampyrus      QQAPQNPPLS SDRMYEYGSI EKVKNDSVNP ELV-----ML GEKNKNPAEQ
R. aegyptiacus   QQAPQNPPLS SDRMYEYGSI EKVKNDSVNP ELL-----IL GEKSQNLAEQ
B. taurus        QQAPQDLALS SDKMYEYGSI EKVKNGYVNQ ELV-----LQ GGKTKNPAEQ
E. caballus      QQDSQDPLLS SDRMYEYGSI EKVKNGYINP EMV-----LQ GEKTTNT-QQ
M. musculus      QQDPQGSSLS ASGMHEYGSI EKVKNGGADT EQP-----VQ EWKNKKPSGQ
H. glaber        QQAPQDPSLS SDRMCKYGST EKAKNGYVNP ELA-----MQ GRKNKSPAEQ
H. sapiens       QQTPQDPPLS SDGMYEYGSI EKVKNGYVNP ELA-----MQ GAKNKNHAEQ
M. domestica     QHNPHSPLLM QNGGSDYGSL ERVKNGFMKT KQTELATAKR GEVMENTKNQ

                 351                                                400
M. rufoniger     VQRTMTMKSL LRALVNMPSH YRCLCISHLI GWTAFLSNML FFTDFMGRIV
M. davidii       IQRTMTMKSL LRALVNMPSH YRCLCISHLI GWTAFLSNML FFTDFMGRIV
M. brandtii      IQKTMTMKSL LRALVNMPSH YRCLCISHLI GWTAFLSNML FFTDFMGRIV
M. lucifugus     IQRTMTMKSL LRALVNMPSH YRCLCISHLI GWTAFLSNML FFTDFMGRIV
E. fuscus        IQRTMTMKSL LRALVNMPSH YRCLCISHLI GWTAFLSNML FFTDFMGRIV
P. alecto        TQRAMTMKSL LRALVSMPSH HRCLCISHLI GWTAFLCNML FFTDFMGQIV
P. vampyrus      TQRAMTMKSL LRALVSMPSH YRYLCISHLI GWTAFLCSML FFTDFMGQIV
```

R. aegyptiacus	TRRTVTLKSL LRALVSMPSH YRCLCISHLI GWTAFLSNML FFTDFMGQIV
B. taurus	TQRTMTLRSL LRALRSMPPH YRCLCISHLI GWTAFLSNML FFTDFMGQIV
E. caballus	TRRTMTMKSL LRALVSMPPH YRYLCISHLL GWTAFLSNML FFTDFMGQIV
M. musculus	SQRTMSMKSL LRALVNMPSH YRCLCVSHLI GWTAFLSNML FFTDFMGQIV
H. glaber	AQRVMTMKSL LRALVNMPPH YRCLCISHLI GWTAFLSNML FFTDFMGQIV
H. sapiens	TRRAMTLKSL LRALVNMPPH YRYLCISHLI GWTAFLSNML FFTDFMGQIV
M. domestica	TQNKMTMRSL LKAILSMPPH YRYLCVSHLI GWTAFLSNML FFTDFMGQIV

	401 450
M. rufoniger	YHGNPYSAHN STEFLIYERG VEVGCWGLCI NAVFSSLYSY FQKALVSYIG
M. davidii	YHGNPYSAHN STEFLIYERG VEVGCWGLCI NAVFSSLYSY FQKALVSYIG
M. brandtii	YHGNPYSAHN STEFLIYERG VEVGCWGLCI NAVFSSLYSY FQKALVSYIG
M. lucifugus	YHGNPYSAHN STEFLIYERG VEVGCWGLCI NAVFSSLYSY FQKALVSYIG
E. fuscus	YHGNPYSAHN STEFLIYERG VEVGCWGLCI NAMFSSLYSY FQKALVSYIG
P. alecto	YHGDPYSAHN STEFLIYERG VEVGCWGLCL NSLFSSLYSY FQKALVSYTG
P. vampyrus	YHGDPYSAHN STEFLIYERG VEVGCWGLCI NSLFSSLYSH FQKALVSYIG
R. aegyptiacus	YHGDPYSAHN STEFLIYERG VEVGCWGLCI NSMFSSLYSY FQKALVSYVG
B. taurus	YHGDPYGAHN STEFLIYQRG VEVGCWGLCI NSMFSSLYSY FQKVLVPCIG
E. caballus	YHGDPYSAHN STEFLIYQRG VEVGCWGLCI NSVFSSLYSY FQKVLVSYVG
M. musculus	YHGDPYGAHN STEFLIYERG VEVGCWGLCI NSVFSSVYSY FQKAMVSYIG
H. glaber	YRGDPYSAHN STEFLIYERG VEVGCWGLCI NSVFSSLYSY FQKALVPYIG
H. sapiens	YRGDPYSAHN STEFLIYERG VEVGCWGFCI NSVFSSLYSY FQKVLVSYIG
M. domestica	YHGDPYAPHN STSFLIYERG VEVGCWGLCI NSVFSSLYSY FQKVLLSYVG

	451 500
M. rufoniger	LKGLYFMGYL LFGLGTGFIG LFPNVYSTLA LCASFGVMSS TLYTVPFNLM
M. davidii	LKGLYFMGYL LFGLGTGFIG LFPNVYSTLA LCASFGVMSS TLYTVPFNLM
M. brandtii	LKGLYFMGYL LFGLGTGFIG LFPNVYSTLA LCASFGVMSS TLYTVPFNLM
M. lucifugus	LKGLYFMGYL LFGLGTGFIG LFPNVYSTLA LCASFGVMSS TLYTVPFNLM
E. fuscus	LKGLYFMGYL LFGLGTGLIG LFPNVYSTLA LCASFGVMSS TLYTVPFNLM
P. alecto	LKGLYFMGYL LFGLGTGLMG LFRNVYSTLA LCASFGVMSS TLSTIPFNLI

```
P. vampyrus      LKGLYFMGYL LFGLGTALMG FFRNVYSTLA LCTLFGVMSS TLCTIPFNLI
R. aegyptiacus   LKGLYFMGYL LFGLGTGFIS LFPNVYSALA LCASFGVMSS TLYTVPFNLI
B. taurus        LKGLYFMGYL LFGLGTGFIG LFPNVYSTLA MCTLFGVMSS TLYTVPFTLI
E. caballus      LKGLYFMGYL LFGLGTGFIG LFPNIYSTLV LCTSFGVMSS TLYTVPFNLI
M. musculus      LKGLYFMGYL LFGLGTGFIG LFPNVYSTLV LCSMFGVMSS TLYTVPFNLI
H. glaber        LKGLYFMGYL LFGLGTGFIG LFPNVYSTLV LCTLFGIMSS TLYTVPFNLM
H. sapiens       LKGLYFTGYL LFGLGTGFIG LFPNVYSTLV LCSLFGVMSS TLYTVPFNLI
M. domestica     LKGLYIMGYL LFGLGTGFIG LFPNVYSTLV LCALFGVMSS TLYTVPFNLM

                 501                                              550
M. rufoniger     AEYHRKEQEE QKQKARAG-- APDSGSRGQG LDCATLTCMV QLAQILVGGG
M. davidii       AEYHREEQEE QKQKARAG-- APDSDGRGQG LDCATLTCMV QLAQILVGGG
M. brandtii      AEYHREEQEE QKQKARAG-- APDSGGRGQG LDCATLTCMV QLAQILVGGG
M. lucifugus     AEYHREEQEE QKQKARAG-- APDSGGRGQG LDCATLTCMV QLAQILVGGG
E. fuscus        AEYHREEQEE Q-QKARAG-- APDSGGRGQG LDCATLTCMV QLAQILVGGG
P. alecto        AEYHREEQEQ QKQQAQEG-- GPACSGRGEG LDCATLTCMV QLAQILVGGG
P. vampyrus      AEYHREEQEQ QKQQAQEG-- GPACSGRGEG LDCATLTCMV QLAQILVGGG
R. aegyptiacus   AEYHREEQEE QKQQAQEA-- DPASRGRGQG LDCATLTCMV QLAQILVGGG
B. taurus        AVYHHEE--- QKQRALGG-- GPDGSSRGQG LDCAALTSMV QLAQILVGGG
E. caballus      AEYHREEQEK QRRQAQGG-- DVDSSGRGQG LDCAALTCMV QLAQILVGGG
M. musculus      AEYHREEEKE KGQ-EAPG-- GPDNQGRGKG VDCAALTCMV QLAQILVGGG
H. glaber        AEYHLAEEKE KRRQAPGWSS DSSGTERGKG VDCATLTCMV QLAQILVGSG
H. sapiens       TEYHREEEKE RQ-QAPGG-- DPDNSVRGKG MDCATLTCMV QLAQILVGGG
M. domestica     AEYHHEDERQ QATG------ MAPSGCRGRG IDCAALTCMV QLAQILVGVG

                 551                                587
M. rufoniger     LGFLVNMVGS VVVVVISASL VALMGCCFVA LFVRYVQ
M. davidii       LGFLVNMAGS VVVVVISASL VALMGCCFVA LFVRYVQ
M. brandtii      LGFLVNMAGS VVVVVISASL VALMGCCFVA LFVRYVQ
M. lucifugus     LGFLVNMAGS VVVVVISASL VALMGCCFVA LFVRYVQ
E. fuscus        LGLLVNMAGS VVVVVISASL VALMGCCFVA LFVRYVQ
```

P. alecto	LGFLVNMAGS VVVVVITASA VALIGCCFVA LFVRCVD
P. vampyrus	LGFLVNMAGS VVVVVITASA VALIGCCFVA LFVRCVD
R. aegyptiacus	LGFLVNMAGS VVVVVITASA VALIGCCFVA LFVRYVD
B. taurus	LGLLVNTAGS VVVVVITASA VALIGCCFVA LFVRYVD
E. caballus	LGFLVNIAGS VVVVVITASV VALIGCCFVV LFVRYVA
M. musculus	LGFLVNMAGS VVVVVITASA VSLIGCCFVA LFVRYVD
H. glaber	LGFLVNTAGS VIVVVITAST VALIGCCFVA VFVRYVG
H. sapiens	LGFLVNTAGT VVVVVITASA VALIGCCFVA LFVRYVD
M. domestica	LGVLVTSAES VVVVVIVASA VALFGCCFVA LFVRCVE

Topological domain: Extracellular

(C)

TYRP1

```
               1                                                  50
M. rufoniger   ---------- ---------- ---------- ---------M EVHKLLSLGY
M. davidii     ---------- ---------- ---------- ---------M EVHKLLSLGY
M. brandtii    ---------- ---------- ---------- ---------M EVHRLLSLGY
M. lucifugus   MTAFDKWHQD NLIFLYVP-- ---QSLHKGP QNQSLASSRM EVHRLLSLGY
E. fuscus      ---------- ---------- ---------- ---------M EVHKLLPLGY
P. alecto      ---------- ---------- ---------- ---------M KDHKFLSLGC
P. vampyrus    ---------- ---------- ---------- ---------M KDNKFLSLGC
R. aegyptiacus ---------- ---------- ---------- ---------M KDHKLLCLGC
B. taurus      MKSIL----- -KLDFYLRAS VSTQRAANQK LSCILASSRM KSPTLLSLGY
E. caballus    ---------- ---------- ---------- ---MFASSRM KAHKLLSLGY
M. musculus    ---------- ---------- ---------- ---------M KSYNVLPLAY
H. glaber      ---------- ---------- ---------- ---------M STPGRPALRP
H. sapiens     ---------- ---------- ---------- ---------M SAPKLLSLGC
M. domestica   ---------- ---------- ---------- ---------- -MARRGHLFW

               51                                                100
M. rufoniger   -IFLPLLFTQ QAGAQFPREC ATTEALRNGV CCPDLAPVSG PGTDPCGSSS
M. davidii     -VFLPLLFTQ QAGAQFPREC ATTEALRNGV CCPDLAPVSG PGTDPCGSSS
M. brandtii    -IFLPLLFTQ QAGAQFPREC ATTEALRNGV CCPDLAPVSG PGTDPCGSSS
M. lucifugus   -VFLPLLFTQ QAGAQFPREC ATTEALRNGV CCPDLAPVSG PGTDPCGSSS
E. fuscus      -IFLPLLFTQ QAGAQFPREC ATTEALRNGV CCPDLSPVSG PGTDPCGSSS
P. alecto      -IFLSLLFFQ QTWAQFPREC ATIEALRNGV CCPDLSALSG PGTDLCGSSS
P. vampyrus    -IFLSLLFFQ QTWAQFPREC ATIEALRNGV CCPDLSALSG PGTDLCGSSS
R. aegyptiacus -IFLPLFFFQ QTWAQFPREC ATIEALRNGV CCPDLSALSG PGTDLCGSSS
B. taurus      -MFLVLLFFQ QAWAQFPREC ATIEALRNGV CCPDLSPLSG PGSDRCGLSS
E. caballus    -LFLPPLFFQ QAWAQFPREC ATVEALKNGV CCPDLNPLSG PGTDRCGSSS
```

M. musculus IS-LFLMLFY QVWAQFPREC ANIEALRRGV CCPDLLPSSG PGTDPCGSSS
H. glaber VLLTLLLLWH LARAQFPRAC ASAPALRNGE CCPDLRPAAG PGSDRCGAAS
H. sapiens -IFFPLLLFQ QARAQFPRQC ATVEALRSGM CCPDLSPVSG PGTDRCGSSS
M. domestica -MLWPLLLLH QSDAQFPREC ATLDALRSGE CCPDLSLGPE PGTDSCGSST

Topological domain: Lumenal, melanosome

101 150
M. rufoniger GRGRCEAVTA DSRPHGPQYP HDGRDDREGW PTRFFNRTCH CNGNFWGHNC
M. davidii GRGRCEAVTA DSRPHGPQYP HDGQDDREGW PTRFFNRTCH CNGNFSGHNC
M. brandtii GRGRCEAVTA DSRPHGPQYP HDGLDDREGW PTRFFNRTCH CNGNFSGHNC
M. lucifugus GRGRCEAVTA DSRPHGPQYP HDGRDDREGW PTRFFNRTCH CNGNFSGHNC
E. fuscus GRGRCEAVSA DFRPHSPQYP HDGRDDREGW PTRFFNRTCH CNGNFSGHNC
P. alecto GRGRCEAVTA DFRPHSPLYP HDGRDDRERW PTRFFNRTCH CNGNFSGHNC
P. vampyrus GRGRCEAVTA DFRPHSPLYP HDGRDDRESW PTRFFTRACH CNGNFSGHNC
R. aegyptiacus GRGRCEAVTA DFRPHSPLYP HDGRDDREGW PTRFFNRTCH CNGNFSGHNC
B. taurus GRGRCEVVIA DSRPHSHHYP HDGRDDREGW PTRFFNRTCH CNGNFSGHNC
E. caballus GRGRCEAVTA DSRPHSHHYP HDGRDDREAW PTRFFNRTCH CNGNFSGHNC
M. musculus GRGRCVAVIA DSRPHSRHYP HDGKDDREAW PLRFFNRTCQ CNDNFSGHNC
H. glaber GRGRCEAVMA DSRPHSAGYP HDGRDDREAW PLRFFNRTCR CAGNFAGHNC
H. sapiens GRGRCEAVTA DSRPHSPQYP HDGRDDREVW PLRFFNRTCH CNGNFSGHNC
M. domestica GRGRCDAVTA DSRPHGPQYP HDGRDDREAW PTRFFNRTCH CNGNFSGYNC

Topological domain: Lumenal, melanosome

151 200
M. rufoniger GTCRPGWRGI ACDQRVLTVR RNLLDFSIEE KNYFVQALDM AKRTTHPQWV
M. davidii GTCRPGWRGT ACDQRVLTVR RNLLDLSTEE KNYFVRALDM AKRTTHPQFV
M. brandtii GTCRPGWRGT ACDQRVLTVR RNLLDLSTEE KNYFVRALDM AKRTTHPQFV
M. lucifugus GTCRPGWRGN ACDQRVLTVR RNLLDLSTEE KNYFVRALDM AKRTTHPQFV
E. fuscus GTCRPGWRGT ACDQRVLTVR RNLLDLSTEE KNYFVRALDM AKRTTHPRFV
P. alecto GTCRPGWRGA ACDERILTVR RNLLDLSTEE KKYFVQALDM AKRTTHPQFV
P. vampyrus GTCRPGWRGA ACDERILTVR RNLLDLSTEE KKYFVQALDM AKRTTHPQFV
R. aegyptiacus GTCRPGWRGA ACDERILTVR RNLLDLSTEE KNYFVQALDM AKRTTHPQFV
B. taurus GTCRPGWGGA ACDQRVLTVR RNLLDLSTEE KNRFVRALDM AKRTTHPQFV

E. caballus GTCRPGWRGA ACDQRVLTVR RNLLDLSTEE KSYFVRALDM AKRTTHPQFV

M. musculus GTCRPGWRGA ACNQKILTVR RNLLDLSPEE KSHFVRALDM AKRTTHPQFV

H. glaber GACRPGWAGP GCSQRALAVR RNLLDLSAEE KNHFVQALDM AKRTTHPQFV

H. sapiens GTCRPGWRGA ACDQRVLIVR RNLLDLSKEE KNHFVRALDM AKRTTHPLFV

M. domestica GTCKPGWRGS TCDQRVITVR RNLLDLSTEE RNRFIQALDM AKRTIHPHLV

Topological domain: Lumenal, melanosome

201 250

M. rufoniger IATRRSEEIL GPDGNTPQFE NISIYNYFVW THYYSVKKTF LGAGQESFGE

M. davidii IATRRSEEIL GPDGNTPQFE NISIYNYFVW THYYSVKKTF LGAGQESFGE

M. brandtii IATRRSEEIL GPDGNTPQFE NISIYNYFVW THYYSVKKTF LGAGQESFGE

M. lucifugus IATRRSEEIL GPDGNTPQFE NISIYNYFVW THYYSVKKTF LGAGQESFGE

E. fuscus IATRRSEEIL GPDGNTPQFE NISIYNYFVW THYYSVKKTF LGAGQESFGE

P. alecto IATRRSEEIL GPDGNTPQFE NISIYNYFVW THYYSVKKTF LGAGQKSFGE

P. vampyrus IATRRSEEIL GPDGNTPQFE NISIYNYFVW THYYSVKKTF LGAGQKSFGE

R. aegyptiacus IATRRSEEIL GPDGNTPQFE NISIYNYFVW THYYSVKKTF LGAGQESFGE

B. taurus IATRRSEEIL GPDGNTPQFE NISIYNYFVW THYYSVKKTF LGAGQESFGE

E. caballus IATRRSEEIL GPDGDTPQFE NVSIYNYFVW THYYSVKKTF LGAGQESFGE

M. musculus IATRRLEDIL GPDGNTPQFE NISVYNYFVW THYYSVKKTF LGTGQESFGD

H. glaber IATRRSKEIL GEDGNTPQFE NISIYNYFVW THYYSVKKTF LGPGQESFGE

H. sapiens IATRRSEEIL GPDGNTPQFE NISIYNYFVW THYYSVKKTF LGVGQESFGE

M. domestica IATRRSEELL GTDGNTPQFE NISIYNYFVW AHYYSVKKTF LGEGQESFGA

Topological domain: Lumenal, melanosome

251 300

M. rufoniger VDFSHEGPAF LTWHRYHLLQ LERDMQEMLQ NPSFSLPYWN FATGKNVCDI

M. davidii VDFSHEGPAF LTWHRYHLLQ LERDMQEMLQ NPSFALPYWN FATGKNVCDI

M. brandtii VDFSHEGPAF LTWHRYHLLQ LERDMQEMLQ NPSFSLPYWN FATGKNVCDI

M. lucifugus VDFSHEGPAF LTWHRYHLLQ LERDMQEMLR NPSFSLPYWN FATGKNVCDI

E. fuscus VDFSHEGPAF LTWHRFHLLQ LERDMQEMLQ NPSFSLPYWN FATGKNVCDI

P. alecto VDFSHEGPAF LTWHRYHLLQ LERDMQEMLR NPSFSLPYWN FATGKNTCDI

P. vampyrus VDFSHEGPAF LTWHRYHLLQ LERDMQEMLR NPSFSLPYWN FATGKNTCDI

R. aegyptiacus VDFSHEGPAF LTWHRYHLLQ LERDMQEMLR NPSFSLPYWN FATGKNTCDI

B. taurus	VDFSHEGPAF	LTWHRYHLLQ	LERDMQEMLQ	DPSFSLPYWN	FATGKNTCDI
E. caballus	VDFSHEGPAF	LTWHRYHLLQ	LERDMQEMLQ	DPSFSLPYWN	FATGRNICDI
M. musculus	VDFSHEGPAF	LTWHRYHLLQ	LERDMQEMLQ	EPSFSLPYWN	FATGKNVCDV
H. glaber	VDFSHEGPAF	LTWHRYHLLQ	LEKDMQDMLQ	EPSFSLPYWD	FATGKNVCDV
H. sapiens	VDFSHEGPAF	LTWHRYHLLR	LEKDMQEMLQ	EPSFSLPYWN	FATGKNVCDI
M. domestica	VDFSHEGPAF	LTWHRYHLLQ	LEKDMQEMLQ	DPTFALPYWN	FATGGNTCDI

Topological domain: Lumenal, melanosome

	301				350
M. rufoniger	CTDDMMGSRS	NFDPNLISPN	SVFSQWRVVC	ESLEDYDTLG	TLCNSTEGAP
M. davidii	CTDDMMGSRS	NFDPNFISPN	SVFSQWRVVC	ESLEDYDTLG	TLCNSTEGAP
M. brandtii	CTDDMMGSRS	NFDPNLISAN	SVFSQWRVVC	ESLEDYDTLG	TLCNSTEGAP
M. lucifugus	CTDDMMGSRS	SFDPNLISAN	SVFSQWRVVC	ESLEDYDTLG	TLCNSTEGAP
E. fuscus	CTDDMMGSRS	NFDPNLISPN	SVFSQWRVVC	ESLEDYDTLG	TLCNSTEGAP
P. alecto	CTDDLMGSRS	NFDSNLISPN	SVFSQWRVVC	ESLEDYDTLG	TLCNSTEGGP
P. vampyrus	CTDDLMGSRS	NFDSNLISPN	SVFSQWRVVC	ESLEDYDILG	TLCNSTEGGP
R. aegyptiacus	CTDDLMGSRS	NFDSNLISPN	SVFSQWRVVC	ESLEDYDTLG	TLCNSTEGGP
B. taurus	CTDDLMGSRS	NFDSTLISPN	SVFSQWRVVC	ESLEDYDTLG	TLCNSTEGGP
E. caballus	CTDDLMGSRS	NFDSSLISPN	SVFSQWRVVC	ESLEDYDTLG	TLCNSTEGGP
M. musculus	CTDDLMGSRS	NFDSTLISPN	SVFSQWRVVC	ESLEEYDTLG	TLCNSTEGGP
H. glaber	CTDDLMGARN	SFDSTLISPN	SVFSQWHMVC	ESLEDYDTLG	TLCNSTEGGP
H. sapiens	CTDDLMGSRS	NFDSTLISPN	SVFSQWRVVC	DSLEDYDTLG	TLCNSTEDGP
M. domestica	CTDNLMGSRS	NFDFSLISPN	SVFSQWRVLC	ESVEDYDTLG	TICNSTEGGP

Topological domain: Lumenal, melanosome

	351				400
M. rufoniger	IRRNPAGNVA	RPMVQRLPEP	QDVAQCLEVG	LFDTPPFYSN	STNSFRNTVE
M. davidii	IRRNPAGNVA	RPMVQRLPEP	QDVAQCLEVG	LFDTPPFYSN	STNSFRNTVE
M. brandtii	IRRNPAGNVA	RPMVQRLPEP	QDVAQCLEVG	LFDTPPFYSN	STNSFRNTVE
M. lucifugus	IRRNPAGNVA	RPMVQRLPEP	QDVAQCLEVG	LFDTPPFYSN	STNSFRNTVE
E. fuscus	IRRNPAGNVA	RPMVQRLPEA	QDVAQCLEVG	LFDTPPFYSN	SSNSFRNTVE
P. alecto	IRRNPAGNVA	RPMVQRLPEP	QDVIQCLEVG	LFDTPPFYSN	STNSFRNTVE
P. vampyrus	IRRNPAGNVA	RPMVQRLPEP	QDVTQCLEVG	LFDTPPFYSN	STNSFRNTVE

```
R. aegyptiacus  IRRNPAGNVA RPMVQRLPEP QDVARCLEVG LFDTPPFYSN STNSFRNTVE
B. taurus       IKRNPAGNVA RPMVQRLPKP QDVAQCLEVG SFDTPPFYSN STNSFRNTVE
E. caballus     IRRNPAGNVA RPMVQRLPEP QDVAQCLEVG LFDTPPFYSN STNSFRNTVE
M. musculus     IRRNPAGNVG RPAVQRLPEP QDVTQCLEVR VFDTPPFYSN STDSFRNTVE
H. glaber       IRRNPAGNVA RPMVQRLPAP QDVAQCLEVG VFDTPPFYSN STDSFRNTVE
H. sapiens      IRRNPAGNVA RPMVQRLPEP QDVAQCLEVG LFDTPPFYSN STNSFRNTVE
M. domestica    IRRNPAGNVA RPMVQRLPEP QDVAQCLEVG LFDTPPFYSN STNSFRNTVE
```

Topological domain: Lumenal, melanosome

```
                401                                              450
M. rufoniger    GYNDPMGRYD PAVRSLHNLA HLFLNGTGGQ THLSPNDPIF ILLHTFTDAI
M. davidii      GYSDPMGRYD PAVRSLHNLA HLFLNGTGGQ THLSPNDPIF VLLHTFTDAI
M. brandtii     GYSDPMGRYD PAVRSLHNLA HLFLNGTGGQ THLSPNDPIF VLLHTFTDAI
M. lucifugus    GYSDPMGRYD PAVRSLHNLA HLFLNGTGGQ THLSPNDPIF VLLHTFTDAI
E. fuscus       GYSDPMGRYD PAVRTLHNLA HLFLNGTGGQ THLSPNDPIF VLLHTFTDAI
P. alecto       GYSDPTGKYD PAVRSLHNLA HLFLNGTGGQ THLSPNDPIF ILLHTFTDAL
P. vampyrus     GYSDPTGKYD PAVRSLHNLA HLFLNGTGGQ THLSPNDPIF VLLHTFTDAL
R. aegyptiacus  DIST------ ---------- ---------- ---------- ----------
B. taurus       GYSHPTGRYD PAVRSLHNLA HLFLNGTGGQ THLSPNDPIF VLLHTFTDAV
E. caballus     GYSDPTGKYD PAVRSLHNLA HLFLNGTGGQ THLSPNDPIF VLLHTFTDAV
M. musculus     GYSAPTGKYD PAVRSLHNLA HLFLNGTGGQ THLSPNDPIF VLLHTFTDAV
H. glaber       GYSDPTGKYD PAVRSLHNLA HLFLNGTGGQ THLSPNDPIF VLLHTFTDAI
H. sapiens      GYSDPTGKYD PAVRSLHNLA HLFLNGTGGQ THLSPNDPIF VLLHTFTDAV
M. domestica    GYSDPTGRYD PAVRSLHNLA HLFLNGTGGQ THLSPNDPIF VLLHTFTDAV
```

Topological domain: Lumenal, melanosome

```
                451                                              500
M. rufoniger    FDEWLRRYNA DISTFPLENA PIGHNRQYNM VPFWPPXTNT EMFVTAPDNL
M. davidii      FDEWLRRYNA DISTFPLENA PIGHNRQYNM VPFWPPITNT EMFVTAPDNL
M. brandtii     FDEWLRRYNA DISTYPLENA PIGHNRQYNM VPFWPPITNT EMFVTAPDNL
M. lucifugus    FDEWLRRYNA DISTFPLENA PIGHNRQYNM VPFWPPVTNT EMFVTAPDNL
E. fuscus       FDEWLRRYNA DISTFPLENA PIGHNRQYNM VPFWPPITNT EMFVTAPDNL
P. alecto       FDEWLRRYNA DISVFPLENA PIGHNRQYNM VPFWPPITNT EMFVTAPDNL
```

P. vampyrus FDEWLRRYNA DISAFPLENA PIGHNRQYNM VPFWPPITNT EMFVTAPDNL

R. aegyptiacus ---------- ----FPLKMP LI-IDNNQIW YAFWPPITNI EMFVTAPDNL

B. taurus FDEWLRRYNA DISTYPLENA PIGHNRQYNM VPFWPPVTNI EMFVTAPDNL

E. caballus FDEWLRRYNA DISTFPLENA PIGHNRQYNM VPFWPPITNV EMFVTAPDNL

M. musculus FDEWLRRYNA DISTFPLENA PIGHNRQYNM VPFWPPVTNT EMFVTAPDNL

H. glaber FDEWLRRYNA DISTFPLENA PIGHNRQFNM VPFWPPVTNT EMFVTAADNL

H. sapiens FDEWLRRYNA DISTFPLENA PIGHNRQYNM VPFWPPVTNT EMFVTAPDNL

M. domestica FDEWLRRYNP DISIFPLENA PIGHNRQYNM VPFWPPVTNT EMFVTAPDNL

Topological domain: Lumenal, melanosome

501 550

M. rufoniger GYTYEVQWPG -RSFHISEII TIAVVAALLL VA---VI--- -FVGASCLIR

M. davidii GYTYEVQWPG -RSFHISEII TIAVVAALLL VA---VI--- -FVGASCLIR

M. brandtii GYTYEVQWPG -RSFRISEII TIAVVAALLL VA---VI--- -FVGASCLIR

M. lucifugus GYTYEVQWPG -RSFRISEII TIAVVAALLL VA---VI--- -FVGASCLIR

E. fuscus GYTYEVQWPG -RSFHISEII TIAVVAALLL VA---VI--- -FVGASCLIR

P. alecto GYTYEVQWPS -RNFSISEIV TIAVIAALLL VA---VI--- -FGGASCLIR

P. vampyrus GYTYEVQWPS -RNFSISEIV TIAVIAALLL VA---VI--- -FGGASCLIR

R. aegyptiacus GYTYEVQWPS -RNFSISEIV TIAVVAALLL VA---VV--- -FVGASCLIR

B. taurus GYTYEVQWPS -RSFSISEIV TIAVVAALSL VA---VI--- -FAGASCLIR

E. caballus GYTYEVQWPG -RDFSISEIV TIAVVAALLV VA---VI--- -FVGASCLIH

M. musculus GYAYEVQWPE APRMKPTSLS SLITINAMLR TMRSSRILTT PWSDSRLPIP

H. glaber GYTYEVQWPT GQNFSISEIV TIAVVAALLL VG---LI--- -FLGISCLIR

H. sapiens GYTYEIQWPS -REFSVPEII AIAVVGALLL VA---LI--- -FGTASYLIR

M. domestica GYAYEVQWPR -RAFNVTEII TMTVVAALIL VA---IV--- -FVVTTCLIR

Topological domain: Lumenal, melanosome

551 596

M. rufoniger ARRNMDEANQ PLLTDQYRHY ---AEEYEK- ----IP-NPX QSMV--

M. davidii ARSNMDEANQ PLLTDQYRHY ---AEEYEK- ----IP-NPN QSMV--

M. brandtii ARSNMDEANQ PLLTDQYRHY ---AEEYEK- ----VP-NPN QSMV--

M. lucifugus ARSNMDEANQ PLLTDQYRHY ---AEEYEK- ----VP-NPN QSMV--

E. fuscus ARSNMDEANQ PLLTDQYRHY ---AEEYEK- ----IP-NPN QSMV--

P. alecto	AKNKRDEANQ	PLLTDQYRHY	---AEEYET-	----IQ-NHN	QSMV--
P. vampyrus	AKNKRDEANQ	PLLTDQYRHY	---AEEYET-	----IQ-NHN	QSMV--
R. aegyptiacus	AKSNMDEANQ	PLLTDQYRDY	---AEEYEK-	----IQ-NPN	QSMV--
B. taurus	ARSNMDEANQ	PLLTDQYQHY	---IEEYEK-	----IH-NPN	QSVV--
E. caballus	ARSNRDEATQ	PLLTDRYQHY	---AAEYEK-	----LP-DPN	QSMV--
M. musculus	ARSRLGS---	NLIETQLSTV	FPLASFFSSQ	DRCGLELKHC	SPVMTV
H. glaber	ARSKMDEASQ	PLLIDQYQHY	---DEGYEK-	----VQ-NPN	QPTV--
H. sapiens	ARRSMDEANQ	PLLTDQYQCY	---AEEYEK-	----LQ-NPN	QSVV--
M. domestica	ARRNKDESNQ	PLLSDQYQRY	---AEEYEK-	----LS-NPS	QSMV--

(D)

OCA2

```
                1                                                   50
M. rufoniger    --------MH LEEEGGRVS- SGKLEVELHQ TSA---SP-- ----------
M. davidii      --------MH LEKEGGRLS- SDKLEVELHQ TSA---SPRA GLGL------
M. brandtii     --------MH LEKEGGRLS- SGKLEVELHQ TSA---SP-- ----------
M. lucifugus    --------MH LEKEGGRLS- SGKLEVELHQ TSA---SPRA GLGL------
E. fuscus       --------MH LEKAGGRPSS GGKLEVELHQ TSA---APRA GLGP------
P. alecto       --------MH LECRDGRLA- SGVLETELHR SSA---PVSA RLGGPEGRAT
P. vampyrus     --------MH LECRDGRLA- SGVLETELHR SSA---PVSA RLGGTEGRAT
R. aegyptiacus  --------MH LERRDGRLS- SGVLETELHR SSA---PASA GLGGPEGRAT
B. taurus       --------MH LENKDGRLA- SGRLEMELHQ TSA---PTSA GLGGSGLVEL
E. caballus     --------MH LESKDGGLT- SGTLEMELHQ TSA---PTCA GLGGPGLVRH
M. musculus     --------MR LENKDIRLA- SAVLEVELHQ TSALSVPTCP DPGRLLTVKP
H. glaber       --------MH LESKDSRAA- PAVQEVELQR TSN---PSGA DPGGLEPPRP
H. sapiens      MREDWQWSMH LEGRDGRRY- PGAPAVELLQ TSV---PSG- ------LAEL
M. domestica    --------MY VENKDGGVI- SRELEMELNQ TSALVHTNVH GHMGAESITD

                51                                                 100
M. rufoniger    ---------- ---------- ---PYPQQAT RQSCWAPVDQ DFGSFLTEQR
M. davidii      AAISRRP--- --QLGGRGX- ------KQAA GQSCWAPVDQ DFGSFLTEQR
M. brandtii     ---------- ---------- ---PYPQQAA GQSCWAPVDQ DFGSFLTEQR
M. lucifugus    AAITRRP--- --QLGVRGAD PQGPYPQQAA GQSCWAPVDQ DFGSFLTEQR
E. fuscus       AASSRRP--- --QLGAGGAD PESPYPREAA GQSCWAPGDQ DCGSFLTEQR
P. alecto       --GSFGP--- --QYGASRTA PPGSYPSAAA QQNFGAPEDQ GLASFPMEQR
P. vampyrus     --GSFGP--- --QYRASKTA PPGSCPSAAA QQNFGAPEDQ GLASFPMEQS
R. aegyptiacus  --GILGP--- --QHGASRAA PPDPCPSAEA KQSFRVPEDQ SFASFLMERR
B. taurus       NTSNRRP--- --QQGVGRAD SLSPYHNGAA GQSCWATVDQ DFGSFLKERR
E. caballus     VMSNCRP--- --QPGVSRAD PPSPCSSRAA RWSHWAPVDQ ELGPFLTEGR
```

M. musculus ATSN------ ---YKLGQAD PCIPYAGEAA GKSVCVPEHT EFGSFLVKGS

H. glaber SSCEH----- ---RG-HGAR LPGPRAGWAA GQSPWTAGRK DVGSFLTERR

H. sapiens VAGKRRL--- --PRGAGGAD PSHSCPRGAA GQSSWAPAGQ EFASFLTKGR

M. domestica NFNELRLLKK FHRIAAVHGE PQESSPSGTE ENDCCIPLDD DFGPLLMQER

101 150

M. rufoniger SHLPFTGFCS SGPRDPCLTE STPLLGGPSQ ERGTRCLPVC HPELITA-ES

M. davidii SRLPFTGFCS SGPGDPCLTE STPLLGRPSQ ERGVRCLPVC HPELITA-ES

M. brandtii SHLPFTGFCS SGPREACCTE STPLLGGPSQ ERGARCLPVY HPELITA-ES

M. lucifugus SHLPFTGFCS SGPREACCTE STPLLGGPSQ ERGARCLPVC HPELITA-ES

E. fuscus ---------- ---------- ---------- ---ARCLPVC RPELITA-ES

P. alecto SHAPFLQAFS -----PSLTE NTPLLRNASQ GKG------- ----------

P. vampyrus SVKSVIATSI -----D---I EL-------- ---DLRIALG STEKFI--LK

R. aegyptiacus SHSPFLQAFS -----PSLTE NTPLLRNASQ GKGSRRMPAG GPEPAGPGAS

B. taurus SHPPFSQVFS SRSKEPCFTE NTPLLRNFSQ EKGSWCMPVC HPEFITGEES

E. caballus SHSPFIQFFS SGSKDLCFTE NTPLLRNSSQ EKGSRCMPVY HPEFIIAEES

M. musculus ---------- SSLKDLSFKE DTPLLWNSSQ KKRSQLMPVH HPEFIATEGS

H. glaber SHSPLPQFLG SRTKDSCFTE NTPLLWNSSQ EKGSHCMPAY HPEFMPEEES

H. sapiens SHSSLPQMSS SRSKDSCFTE NTPLLRNSLQ EKGSRCIPVY HPEFITAEES

M. domestica SHFPSSKFFN SRSKDNCFTE KTPLLKTFSE ENGLQCRTIP HSDFIMEDES

151 200

M. rufoniger WDLSSAKWEG TSLLGGEVAG SSRSTSSDRG DV-------- ---LEGAPVP

M. davidii WDLSSAEWEG TSLLGSEATG SSRSASSDRG DL-------- ---LEGAPV-

M. brandtii WDLSSAEWEG TSLLGSEAAG SSRSASSDRG DL-------- ---LEGAPVP

M. lucifugus WDLSSAEWEG TSLLGSEAAG SSRSASSDRG DL-------- ---LEGAPVP

E. fuscus WDLGSAEWEG RSLLGSEAAG SSRSASSDRG DL-------- ---LEGAPVP

P. alecto ---------- ---------- ---------- ---------- ----------

P. vampyrus YD-------- ---------- ---------- --------VS CGF-------

R. aegyptiacus WESGCAEWDA DARALPVGVP GRRGRTPPQG RCAPRLQPRV CSWKRLLAGQ

B. taurus WEDSSTEWEG RSLLSRELAG SSESAFLEKG EL-------- ---LDSAHIR

```
E. caballus     WENSSAEWEP RSLLSRESTG SSGSTSLEKG EL-------- ---LDGAFIR
M. musculus     WENGLTAWEQ KCMLGKEVAD LSALASSEKR DL-------- ---AGSVHLR
H. glaber       WENSLAEWEP RCTLTGEGAG WSTPASSHKE EP-------- ---TEGTHLR
H. sapiens      WEDSSADWER RYLLSREVSG LSASASSEKG DL-------- ---LDSPHIR
M. domestica    WDSTSSEWEQ RCLLGNEIVS FSTSASSEKS EF-------- ---LDSFHVK

                201                                              250
M. rufoniger    FRLSKLRRCA RRLKVTGLFV FVVMFSVSFS LYPDEGKLWQ LLAVSPLESY
M. davidii      ---------- ---------- -------SFS LYPDEGKLWQ LLAVSPLESY
M. brandtii     FRLSKLRRCA RRLKVTGLFV FVVMCSVSFS LYPDEGKLWQ LLAVSPLESY
M. lucifugus    FRLSKLRRCA RRLKVTGLFV FVVMCSVSFS LYPDEGKLWQ LLAVSPLESY
E. fuscus       FRLSKLSPCW SL-------- -----QVSFS LYPDEGRLWQ LLAVSPLESY
P. alecto       -------RCM QWLKVTGLFV FVVVCTVSFS LYPDQGKSWQ LVAVSQLESY
P. vampyrus     ---------- ---ATYG--- -YYYVEVSFS LYPDQGKSWQ LVAVSQLESY
R. aegyptiacus  ARPKASRRCV QWLKVTGLFV FVVVCTVSFS LYPDQGKSWQ LVAVSQLESY
B. taurus       FRLAKLRCCV QWLKVTGLFV FVVLCSILFS LYPDQGKLWQ LLAVSPLESY
E. caballus     FRLSKLRRCV QWLKVAGLFA FVVVCSILFS LYPDQGKFWQ LLAVSPLESY
M. musculus     AQVSKLGCCV RWIKITGLFV FVVLCSILFS LYPDQGKFWQ LLAVSPLENY
H. glaber       VPVLKLKCCA RWLKVAGLFV FVVLCSILFS LYPDQGKSWQ LLAVSPLESY
H. sapiens      LRLSKLRRCV QWLKVMGLFA FVVLCSILFS LYPDQGKLWQ LLALSPLENY
M. domestica    FNLSKLRSCL RCLKVTGLFI FGVVCSILFS TYPDHGKSWQ MLAVSPLESY
                                          Topological domain: Extracellular
                251                                              300
M. rufoniger    SVNLSSRSDS TLLQVDLAGA LVAGGSSRPG REEHVVVEVT QASAPGSR-R
M. davidii      SANLSSRSDS TLLQVDLAGA LMPGSPSRPG REEHVVVEVT QAGAPSSR-R
M. brandtii     SANLSSRSDS TLLRVDLAGA LVAGGPSRPG REEHVVVEVT QAGAPGSR-R
M. lucifugus    SANLSSRSDS TLLRVDLAGA LVAGGPSRPG REERVVVEVT QAGAPGSR-R
E. fuscus       SANLSSRSDS TLLRVDLAGA LVAGGPSRPG REEHVVVEVT QASAPGSR-R
P. alecto       SANLSGHPEL VTWGLSLT-- ---------- ---------- ----------
P. vampyrus     SANLSGHADS TLLQVDLAGA LAAGGPR-PE SAERVVVEVT RTDAPGSRRR
R. aegyptiacus  SANLSGHVDS TLLQVDLAGA LAAGGPG-PE NAERVVVEVT WTDAPGSRRR
```

B. taurus	SVNLSSHADS MLLQVGLAGA LVAGSPSHLG REEHVMVEVT QANTPGSRRR
E. caballus	SVNLSSHVDS ALLQVDLAGA LVAGSSSRPG REEHIVVEVT QVDAPGSRWR
M. musculus	SVNLSGHADS MILQLDLAGA LMAGGPSGSG KEEHVVVVVT QTDAAGNRRR
H. glaber	SVNLSGHADS TLLQLNLAGA LAASGPSRSG REEHVVVEVT QRSALNSRWQ
H. sapiens	SVNLSSHVDS TLLQVDLAGA LVASGPSRPG REEHIVVELT QADALGSRWR
M. domestica	SVNLTDVGDS ALLKLELAGS LIAGVVNRPQ SEEYIVVEVI LREELGFRRR

Topological domain: Extracellular

	301 350
M. rufoniger	RPQ-QVTHNW TVFLNPRRSE HVVVSRTFEV PSRDGVSISV RAALQQPRAV
M. davidii	WPQ-QVTHNW TVFLDPRRSE HVVVSRTFEV PSRDGVSISV RAALQQPRAV
M. brandtii	RPQ-QVTHNW TVFLDPRRSE HVVVSRTFEV PSRDGVSISV RAALQQPRAV
M. lucifugus	RPQ-QVTHNW TVFLNPRRSD HVVVSRTFEV PSRDGVSISV RAALQQPRAV
E. fuscus	RPQ-QVTHNW TVFLSPRRGE HVVASRTFEV PSRDGVSISV RAALQQPRAV
P. alecto	---------- ---------- ---RAKAVAL LCRETVSIRI RASLQQTQLV
P. vampyrus	RPQ-QVTQNW TVFLNPRRRD RLVVSRIFEV PSRETVSIRI RASLQQTQLV
R. aegyptiacus	RPQ-QATQNW TVFLNPRRSD RLVVSRTFEV PSRETVSIRI RASLQQTQLV
B. taurus	RPQ-QVTHNW TIFLNPSGSE HVVVSRTFEV LSREPVSISI RASLQQTQIV
E. caballus	RPQ-QVTHNW TIFLNPRRNE RSVVSKTFEV LSRDTVSISI RASLQQTPAI
M. musculus	RPQ-QLTYNW TVLLNPR-SE HVVVSRTFEI VSREAVSISI QASLQQTRLV
H. glaber	QPQ-QVTHNW TVLLNPRRSE HLEVSRTFTI LSREAVSIRV RTSFPQTRVV
H. sapiens	RPQ-QVTHNW TVYLNPRRSE HSVMSRTFEV LTRETVSISI RASLQQTQAV
M. domestica	RPQQQVIYNW TIFLNSRRNE RAVTSRTFEI ITRETISINI QAFLQENKVI

Topological domain: Extracellular

	351 400
M. rufoniger	PLLLAHHHLR ASVEAQVTAA AAILAGVYVL IIFEIVHRTL AAMLGSLAAL
M. davidii	PLLLAHQHLR ASVEAQVTAA AAILAGVYVL IIFEIVHRTL AAMLGSLAAL
M. brandtii	PLLLAHQHLR ASVEAQVTAA AAILAGVYVL IIFEIVHRTL AAMLGSLAAL
M. lucifugus	PLLLAHQHLR ASVEAQVTAA AAILAGVYVL IIFEIVHRTL AAMLGSLAAL
E. fuscus	PLLLAHQHLR ASVEAQVTMA AVILAGVYVL IIFEIVHRTL AAMLGSLAAL
P. alecto	PLLMAHQHLR ASVGAQVTAA AAILAGVYAL IIFEIVHRTL AAMLGALAAL
P. vampyrus	PLLLAHQHLR ASVGAQVTAA AAILAGVYAL IIFEIVHRTL AAMLGALAAL

```
R. aegyptiacus  PLLMAHQHLR ASVGAQVTAA AAILAGVYAL IIFEIVHRTL AAMLGSLAAL
B. taurus       PLLMTHQYLR ASVEAQVTIA AVILAGVYVL IIFEIVHRTL AAMLGSLSAL
E. caballus     PLLMAHQYLR ASVEAQVTIA AVILAGVYVL IIFEIVHRTL AAMLGSLAAL
M. musculus     PLLLAHQFLG ASVEAQVASA VAILAGVYTL IIFEIVHRTL AAMLGALAAL
H. glaber       PLLLSLQYLR ISFEAQVTMA AAILAGVYTL IIFEIVHRTL AAMLGALAAL
H. sapiens      PLLMAHQYLR GSVETQVTIA TAILAGVYAL IIFEIVHRTL AAMLGSLAAL
M. domestica    PLSMTHQYLH ADIETQVTIA SVILAGVYML IIFEIVHRTL AAMLGSLAAL
```

Topological domain: Extracellular

```
                401                                              450
M. rufoniger    AALAVTGDRP SFTQVVEWID FETLALLFGM MILVAIFSET GFFDYCAVKA
M. davidii      AALAVTGDRP SLTHVVEWID FETLALLFGM MILVAIFSET GFFDYCAVKA
M. brandtii     AALAVTGDRP SLTHVVEWID FETLALLFGM MILVAIFSET GFFDYCAVKA
M. lucifugus    AALAVTGDRP SLNPRGEWID FETLALLFGM MILVAIFSET GFFDYCAVKA
E. fuscus       AALAVTGDRP SLTHVVEWID FETLALLFGM MILVAIFSET GFFDYCAVKA
P. alecto       AVLAVMGDRP SLARVVEWID FETLALLFGM MVLVAIFSET GFFDYCAVKA
P. vampyrus     AVLAVMGDRP SLARVVEWID FETLALLFGM MVLVAIFSET GFFDYCAVKA
R. aegyptiacus  AALAVMGDRP SLARVVEWID FETLALLFGM MILVAIFSET GFFDYCAVKA
B. taurus       AALAVIGDRP SLTHVVEWID FETLALLFGM MILVAIFSET GFFDYCAVKV
E. caballus     AVLAVIGDRP SLTHVVEWID FETLALLFGM MILVAIFSET GFFDYCAVKA
M. musculus     AALAVVGDRP SLTHVVEWID FETLALLFGM MILVAVFSET GFFDYCAVKA
H. glaber       AALAVIGDRP SLTQVVEWID FETLTLLFGM MILVAIFSET GFFDYCAVKA
H. sapiens      AALAVIGDRP SLTHVVEWID FETLALLFGM MILVAIFSET GFFDYCAVKA
M. domestica    AALAVIGDRP SLVRVVEWID YETLALLFGM MILVAIFSET GFFDYCAVKA
```

Topological domain: Extracellular

```
                451                                              500
M. rufoniger    YRLSRGRVWA MIIMLCLFAA VLSAFLDNVT TALLFTPVTI RLCEVLNLDP
M. davidii      YRLSRGRVWA MIIMLCLFAA VLSAFLDNVT TSLLFTPVTI RLCEVLNLDP
M. brandtii     YRLSRGRVWA MIIMLCLFAA VLSAFLDNVT TSLLFTPVTI RLCEVLNLDP
M. lucifugus    YRLSRGRVWA MIIMLCLFAA VLSAFLDNVT TSLLFTPVTI RLCEVLNLDP
E. fuscus       YRLSRGRVWA MIIMLCLIAA VLSAFLDNVT TSLLFTPVTI RLCEVLNLDP
P. alecto       YRLSRGRVWA MTIMLCLVAA TLSAFLDNVT TSLLFTPVTI RLCEVLNLDP
```

P. vampyrus	YRLSRGRVWA	MTIMLCLVAA	TLSAFLDNVT	TSLLFTPVTI	RLCEVLNLDP
R. aegyptiacus	YRLSRGRVWA	MTIMLCLVAA	TLSAFLDNVT	TSLLFTPVTI	RLCEVLNLDP
B. taurus	YQLSRGRVWT	MIFMLCLVAA	VLSAFLDNVT	TVLLFTPVTI	RLCEVLNLDP
E. caballus	YQLSRGRVWA	MIIMLCLIAA	ILSAFLDNVT	TSLLFTPVTI	RLCEVLNLDP
M. musculus	YQLSRGRVWA	MIFMLCLMAA	ILSAFLDNVT	TMLLFTPVTI	RLCEVLNLDP
H. glaber	YRLSRGRVWA	MIIMLCLIAA	VLSAFLDNVT	TMLLFTPVTI	RLCEVLNLDP
H. sapiens	YRLSRGRVWA	MIIMLCLIAA	VLSAFLDNVT	TMLLFTPVTI	RLCEVLNLDP
M. domestica	YRLSRGRVWA	MIIILCLFAA	FLSAFLDNVT	TMLLFTPVTI	RLCEVLNLDP

	501				550
M. rufoniger	RQVLIAEVIF	TNIGGAATAI	GDPPNVMIVS	NQELRKTGLD	FAG--FTAHM
M. davidii	RQVLIAEVIF	TNIGGAATAI	GDPPNVMIVS	NQELRKMGLD	FAG--FTAHM
M. brandtii	RQVLIAEVIF	TNIGGAATAI	GDPPNVMIVS	NQELRKMGLD	FAG--FTAHM
M. lucifugus	RQVLIAEVIF	TNIGGAATAI	GDPPNVMIVS	NQELRKMGLD	FAG--FTAHM
E. fuscus	RQVLIAEVIF	TNIGGAATAI	GDPPNVIIIS	NQELRKMGLD	FAG--FTAHM
P. alecto	RHVLIAEVIF	TNIGGAATAI	GDPPNVIIVS	NQELREAGLD	FAS--FTAHM
P. vampyrus	RHVLIAEVIF	TNIGGAATAI	GDPPNVIIVS	NQELREAGLD	FAS--FTAHM
R. aegyptiacus	RHVLIAEVIF	TNIGGAATAI	GDPPNVIIVS	NQELREAGLD	FAG--FTAHM
B. taurus	RQVLIAEVIF	TNIGGAATAI	GDPPNVLIVS	NQELRKMVCI	TIIRLFPINI
E. caballus	RQVLIAEVIF	TNIGGAATAI	GDPPNVIIVS	NQELRKMGLD	FAG--FTAHM
M. musculus	RQVLIAEVIF	TNIGGAATAI	GDPPNVIIVS	NQELRKMGLD	FAG--FTAHM
H. glaber	RQVLIAEVIF	TNIGGAATAI	GDPPNVIIVS	NQELRKMGLD	FAR--FTAHM
H. sapiens	RQVLIAEVIF	TNIGGAATAI	GDPPNVIIVS	NQELRKMGLD	FAG--FTAHM
M. domestica	RHVLIAEVIF	TNIGGAATAV	GDPPNVIIVS	NQELRKTGMD	FAG--FTAHM

	551				600
M. rufoniger	FVGICFVLLF	SF--PLLRLL	YWN-------	----------	----------
M. davidii	FVGICFVLLF	SF--PLLRLL	YWN-------	----------	----------
M. brandtii	FVGICFVLLF	SF--PLLRLL	YWN-------	----------	----------
M. lucifugus	FVGICFVLLF	SF--PLLRLL	YWN-------	----------	----------
E. fuscus	FVGICFVLLF	SF--PLLRLL	FWN-------	----------	----------

```
P. alecto       FVGICLVLLF CF--PLLRLL YWN------- ---------- ----------
P. vampyrus     FVGICLVLLF CF--PLLRLL YWN------- ---------- ----------
R. aegyptiacus  FVGICFVLLF CV--PLLRLL YWN------- ---------- ----------
B. taurus       EPSL-LAHLV TFYQHGYHFL LWSVLGHLES GRLGRQHAIQ TKEDPELRDH
E. caballus     FVGICFVLLF SF--PLLRLL YWN------- ---------- ----------
M. musculus     FLGICLVLLV SF--PLLRLL YWN------- ---------- ----------
H. glaber       FVGICLVLLV SF--PLLRLL YWN------- ---------- ----------
H. sapiens      FIGICLVLLV CF--PLLRLL YWN------- ---------- ----------
M. domestica    FIGICLILLV SF--PFLRLL YCN------- ---------- ----------
```

Topological domain: Extracellular

```
                601                                             650
M. rufoniger    ---------- ---------- ---------- ---------R KLYNKEPSEI
M. davidii      ---------- ---------- ---------- ---------R KLYNKEPSEI
M. brandtii     ---------- ---------- ---------- ---------R KLYNKEPSEI
M. lucifugus    ---------- ---------- ---------- ---------R KLYNKEPSEI
E. fuscus       ---------- ---------- ---------- ---------R KLYNKEPSEI
P. alecto       ---------- ---------- ---------- ---------E KLYNKEPSDI
P. vampyrus     ---------- ---------- ---------- ---------E KLYNKEPSDI
R. aegyptiacus  ---------- ---------- ---------- ---------K KLYNKEPSEV
B. taurus       MSHNHSHMGK HRKHLGGQAM RVACITTGST WTAVTQGTLG KLLKPQLSFP
E. caballus     ---------- ---------- ---------- ---------K KLYNKEPSEI
M. musculus     ---------- ---------- ---------- ---------K KLYNKEPSEI
H. glaber       ---------- ---------- ---------- ---------K KLYNKEPSEI
H. sapiens      ---------- ---------- ---------- ---------R KLYNKEPSEI
M. domestica    ---------- ---------- ---------- ---------K KLYNKEPSEI
```

Topological domain: Extracellular

```
                651                                             700
M. rufoniger    VELKHESVSV ---------- ---------- ---------- -------FHF
M. davidii      VGECAS---- ---------- ---------- ---------- ------SFCF
M. brandtii     VELKHESVSF ---------- ---------- ---------- -------FHF
M. lucifugus    VELKHEIHVW RLTAQRISPA SREETAVRGL LLGKVLALER LLARRLHTFH
```

```
E. fuscus        VELKHEIHVW RLTAQRISPA SREETAVRGL LLGKVLALER LLARRLHSFH
P. alecto        VELKHEIHVW RLTAQRISPA SREETAVRGL LLQKVLALEH LLARRLRTFH
P. vampyrus      VELKHEIHVW RLTAQRISPA SREETAVRGL LLHKVLALEH LLARRLRTFH
R. aegyptiacus   VELKHEIHVW RLTAQRISPA SREETAVREL LLRKVLALEH LLARRLRTFH
B. taurus        AELKHEIHVW RLTAQHISPA SREETAVRGM LLEKVLTLEH LLAQRLHSFH
E. caballus      VELKHEIHVW RLTAQRISPA SREETAVRDL LLGKVLALER LLACRLRTFH
M. musculus      VELKHEIHVW RLTAQRISPA SREETAVRGL LLEKVLALEH LLAQRLHTFH
H. glaber        VELKHEIHVW RLTAQRISPA SREETAVRSL LLQKVLALEH LLARRLHTFR
H. sapiens       VELKHEIHVW RLTAQRISPA SREETAVRRL LLGKVLALEH LLARRLHTFH
M. domestica     VELKHEIHVW KLTAQRINPA SREETAVKCL LMQKVLTLEF LLKKKLKTFQ
```

Topological domain: Extracellular

```
                 701                                              750
M. rufoniger     RQISQEDKNW ETNIQELQKK HRIADRVLLA KCLMVLGFVI CMFFLNSFVL
M. davidii       RQISQEDKNW ETNIQELQKK HRIADRVLLA KCLMVLGFVI CMFFLSSFVP
M. brandtii      RQISQEDKNW ETNIQELQKK HRISDRVLLA KCLMVLGFVI CMFFLNSFVP
M. lucifugus     RQISQEDKNW ETNIQELQKK HRISDRVLLA KCVMVLGFVI CMFFLNSFVP
E. fuscus        RQISQEDKNW ETNIQELQKK HRISDGILLA KCLVVLGFVI FMFFLNSFVP
P. alecto        RQISREDKNW ETNIQELQRK HRVSDGVLLA KCLLVLGLVI LMFFFSSFVP
P. vampyrus      RQISREDKNW ETNIQELQRK RRKRI----- ---------- --------CT
R. aegyptiacus   RQISREDKNW EANIQELQRK HRISDGALLA KCLLVLGLVI LMFFLSSLVP
B. taurus        RQISQEDKNW ETNIQELQRK HRISDKILLA KCLTVLVFVI FMFFLNSFVP
E. caballus      RQISQEDKNW ETNIQELQKK HRISNRILLA KCLMVLGFVI FMFFLNSFVP
M. musculus      RQISQEDKNW ETNIQELQRK HRISDRSLLV KCLTVLGFVI SMFFLNSFVP
H. glaber        RQISQEDKNW ETNIQELQRK HRISDRILLV KCLTVLGFVI FTFFLSSFVP
H. sapiens       RQISQEDKNW ETNIQELQKK HRISDGILLA KCLTVLGFVI FMFFLNSFVP
M. domestica     RQISQEDKNW ETNIQELQKK HRIADKILLI KCLTVLGFVI FMFFLNSFVP
```

Topological domain: Extracellular

```
                 751                                              800
M. rufoniger     AVHLDLGWIA MLGAIWLLIL ADIHDFEVIL HRVEWATLLF FAALFVLMEA
M. davidii       AVHLDLGWIA ILGAIWLLIL ADVHDFEVIL HRVEWATLLF FAALFVLMEA
M. brandtii      AVHLDLGWIA ILGAVWLLIL ADVHDFEVVL HRVEWATLLF FAALFVLMEA
```

```
M. lucifugus    AVHLDLGWIA ILGAIWLLIL ADVHDFEVVL HRVEWATLLF FAALFVLMEA
E. fuscus       AVHLDLGWIA ILGAIWLLIL ADIHDFEIIL HRVEWATLLF FAALFVLMEA
P. alecto       GVHLGLGWIA VLGAIWLLIL ADIHEFEIVL HRVEWATLLF FAALFVLMEA
P. vampyrus     RISRSPGWIA VLGAIWLLIL ADIHEFEIVL HRVEWATLLF FAALFVLMEA
R. aegyptiacus  GVHLGLGWIA VLGAIWLLIL ADIHEFEIIL HRVEWATLLF FAALFVLMEA
B. taurus       GVHLDLGWIA ILGAIWLLIL ADIHDFEIIL HRVEWATLLF FAALFVLMEA
E. caballus     GVHLDLGWIA ILGAIWLLIL ADIHDFEIIL HRVEWATLLF FAALFVLMEA
M. musculus     GIHLDLGWIA ILGAIWLLIL ADIHDFEIIL HRVEWATLLF FAALFVLMEA
H. glaber       GIHLDLGWIA ILGAIWLLIL AEVHDFEIIL HRVEWATLLF FAALFVLMEA
H. sapiens      GIHLDLGWIA ILGAIWLLIL ADIHDFEIIL HRVEWATLLF FAALFVLMEA
M. domestica    GIHLDLGWIA ILGAIWLLVL ADIHDFEIIL HRVEWATLLF FAALFVLMEA
                  Transmembrane: Helical

                801                                                850
M. rufoniger    LGHLHLIEYV GEQTTSLIKM VPEDRRLAAA IVLVVWVSAI ASSLIDNIPF
M. davidii      LGHLHLIEYV GEQTTSLIKM VPEDQRLAAA IVLVVWVSAI ASSLIDNIPF
M. brandtii     LGHLHLIEYV GEQTTSLIKM VPEDRRLAAA IVLVVWVSAI ASSLIDNIPF
M. lucifugus    LGHLHLIEYV GEQTTSLIKM VPEDRRLAAA IVLVVWVSAI ASSLIDNIPF
E. fuscus       LGHLHLIEYV GEQTTSLIKM VPEDRRLAAA IVLVVWVSAI ASSLIDNIPF
P. alecto       LAHLHLIEYV GEQTTSLIKM VPEDRRLAAA IVLVLWVSAT ASSLIDNIPF
P. vampyrus     LAHLHLIEYV GEQTTSLIKM VPEDRRLAAA IVLVLWVSAT ASSLIDNIPF
R. aegyptiacus  LAHLHLIEYV GEQTTLLIKM VPEDRRLAAA IVLVLWVSAA ASSLIDNIPF
B. taurus       LAHLHLIEYV GEQTALLIKM VPEDQRLTAA IILVVWVSGI TSSLIDNIPF
E. caballus     LAHLHLIEYV GEQTALLIKM VPEDRRLAAA IILVIWVSAI ASSLIDNIPF
M. musculus     LTHLHLVEYV GEQTALLIKM VPEDQRFAAA IVLIVWVSAL ASSLIDNIPF
H. glaber       LAHLHLIEYV GEQTALLIKM VPEDRRLAAA MVLVMWVSAL ASSLIDNIPF
H. sapiens      LAHLHLIEYV GEQTALLIKM VPEEQRLIAA IVLVVWVSAL ASSLIDNIPF
M. domestica    LAHLHLIEYI GEQTALLIKM VPEDQRLTAA IILVVWISAL ASSLIDNIPF

                851                                                900
M. rufoniger    TATM------ --------IP VLLNLSQDPE VSLPAPPLMY ALALGACLGG
M. davidii      TATM------ --------IP VLLNLSQDPE VSLPAPPLMY ALALGACLGG
```

```
M. brandtii     TATM------ --------IP VLLNLSQDPE VSLPAPPLMY ALALGACLGG
M. lucifugus    TATM------ --------IP VLLNLSQDPE VSLPAPPLMY ALALGACLGG
E. fuscus       TATM------ --------IP VLLNLSQDPE VSLPAPPLMY ALALGACLGG
P. alecto       TATM------ --------VP VLLNLSRDPE VSLPAPPLMY ALAFGACLGE
P. vampyrus     TATM------ --------KS PP-------S QALSVT---- ----------
R. aegyptiacus  TATM------ --------VP VLLSLSRDPE VRLPTPPLMY ALALGACLGG
B. taurus       TATM------ --------IP VLLNLSRDPE VSLPAPPLMY ALALGSCLGG
E. caballus     TATM------ --------IP VLLNLSQDPE VSLPAPPLMY ALALGACLGG
M. musculus     TATM------ --------IP VLLNLSQDPE ISLPALPLMY ALALGACLGG
H. glaber       TATM------ --------IP VLLSLSQDPG VSLPALPLMY ALALGACLGG
H. sapiens      TATMIDTSYL RIHQLVLKIP VLLNLSHDPE VGLPAPPLMY ALAFGACLGG
M. domestica    TATM------ --------IP VLLNLSQDPE VSLPVKPLIF SLAIGACLGG

                901                                              950
M. rufoniger    NGTLIGASA- ---------- ---------- ---------- ---NVVCAG-
M. davidii      NGTLIGASA- ---------- ---------- ---------- ---NVVCAG-
M. brandtii     NGTLIGASA- ---------- ---------- ---------- ---NVVCAG-
M. lucifugus    NGTLIGASA- ---------- ---------- ---------- ---NVVCAG-
E. fuscus       NGTLIGASA- ---------- ---------- ---------- ---NVVCAG-
P. alecto       LFVVVDAQVF VGQKQTENRL NVHWQNVETE KDGHGPEHAR RPRRVICRRG
P. vampyrus     ---------- ---------- ---------- ---------- ----------
R. aegyptiacus  NGTLIAASA- ---------- ---------- ---------- ---NVVCAG-
B. taurus       NGTLIGASA- ---------- ---------- ---------- ---NVVCAG-
E. caballus     NGTLIGASA- ---------- ---------- ---------- ---NVVCAG-
M. musculus     NGTLIGAST- ---------- ---------- ---------- ---NVVCAG-
H. glaber       NGTLIGASA- ---------- ---------- ---------- ---NVVCAG-
H. sapiens      NGTLIGASA- ---------- ---------- ---------- ---NVVCAG-
M. domestica    NGTLIGASA- ---------- ---------- ---------- ---NVVCAG-

                951                                             1000
M. rufoniger    ---------- ---------- ---------- ---------- ----------
```

```
M. davidii      ---------- ---------- ---------- ---------- ----------
M. brandtii     ---------- ---------- ---------- ---------- ----------
M. lucifugus    ---------- ---------- ---------- ---------- ----------
E. fuscus       ---------- ---------- ---------- ---------- ----------
P. alecto       GPGGTRRPSD LSSPFGQLSF VLGDGPAPPE PRLPSSFLTL AGATRSQITI
P. vampyrus     ---------- ---------- ---------- ---------- ----------
R. aegyptiacus  ---------- ---------- ---------- ---------- ----------
B. taurus       ---------- ---------- ---------- ---------- ----------
E. caballus     ---------- ---------- ---------- ---------- ----------
M. musculus     ---------- ---------- ---------- ---------- ----------
H. glaber       ---------- ---------- ---------- ---------- ----------
H. sapiens      ---------- ---------- ---------- ---------- ----------
M. domestica    ---------- ---------- ---------- ---------- ----------

                1001                                           1046
M. rufoniger    --IAEQHGYG FSFMQFFRLG FPMMVVSCMV ATCYLLVVHV VMGWN-
M. davidii      --IAEQHGYG FSFVQFFRLG FPMMVVSCMV AMCYLLVVHV VMGWN-
M. brandtii     --IAEQHGYG FSFMQFFRLG FPMMVVSCMV AMCYLLVVHV VMGWS-
M. lucifugus    --IAEQHGYG FSFMQFFRLG FPMMVVSCTV AMCYLLVVHV VMGWS-
E. fuscus       --IAEQHGYG FSFVEFFRLG FPMMVVSCMV AMCYLLVVHV VMGWN-
P. alecto       GYPPSQALSV TPGQFLFWLG FPMVVVSCTV GMCYLLVVHV VMGWS-
P. vampyrus     ---------- -PGQFLFWLG FPMVVVSCTV GMCYLLVVHV VMGWS-
R. aegyptiacus  -I-AEQRGYG FSFLQFFRLG FPMVIVSCTV GMCYLLVVHV VMGWS-
B. taurus       --IAEQHGYG FSFMEFFRLG FPMMVVSCMV GMCYLLVAHI VMGWN-
E. caballus     --IAEQHGYG FSFMEFFRLG FPMMVVSCTV GMCYLLVAHV VLGWN-
M. musculus     --IAEKHGYG FSFMEFFRLG FPVMLMSCTI GMCYLLIAHI VVGWN-
H. glaber       --IAEQHGYG FSFMEFFRLG FPMMIVSCTV GMCYLLVVHV VMGWN-
H. sapiens      --IAEQHGYG FSFMEFFRLG FPMMVVSCTV GMCYLLVAHV VVGWN-
M. domestica    --IAEQHGYG FSFMEFFRLG FPMMLVSCTI GMCYLLVAHV VVGWNS
                                          Transmembrane: Helical
```
