# Supplementary material for: Myotis rufoniger genome sequence and analyses: M. rufoniger’s genomic feature and the decreasing effective population size of Myotis bats
Source: PLoS One. 2017 Jul 5;12(7):e0180418. doi: 10.1371/journal.pone.0180418 (PMC5498047; doi:10.1371/journal.pone.0180418)
Supplement: S5 Fig — M. rufoniger specific amino acid sequence changes within N6AMT1 gene are highlighted in yellow. (PDF) [file pone.0180418.s007.pdf]

N6AMT1

```
                 1                                                  50
M. rufoniger     ---------- ---------- ---------- ---------- ----------
M. davidii       ---------- ---------- ---------- ---------- ----------
M. brandtii      ---------- ---------- ---------- ---------- ----------
M. lucifugus     MGVPAAARVP EGPEARSAGQ VTPEAVTALP GSRSEGAGVR VSVPELRALR
E. fuscus        ---------- ---------- ---------- ---------- ----------
P. alecto        ---------- ---------- ---------- ---------- ----------
P. vampyrus      ---------- ---------- ---------- ---------- ----------
R. aegyptiacus   ---------- ---------- ---------- ---------- ----------
B. taurus        ---------- ---------- ---------- ---------- ----------
E. caballus      ---------- ---------- ---------- ---------- ----------
M. musculus      ---------- ---------- ---------- ---------- ----------
H. glaber        ---------- ---------- ---------- ---------- ----------
H. sapiens       ---------- ---------- ---------- ---------- ----------
M. domestica     ---------- ---------- ---------- ---------- ----------

                 51                                                100
M. rufoniger     ---------- -------MPS SGLAGVYPYY QGTQVHATXE DTFLLLDXLE
M. davidii       ---------- ---------- ---------- ---------- ----------
M. brandtii      ---------- ---------- ---------- ---------- ----------
M. lucifugus     AAFPGGLSAP APPNSLPRGS ATRPG---SG AAPPTPYPE- --------AP
E. fuscus        ------MAAP ----SLPTPE HGHVG---RG AFREVYAPAE DTFLLLDALE
P. alecto        ---------- MAAPRFPTPL HGHVG---RG AFNDVYEPAE DTFLLLDALE
P. vampyrus      ---------- MAAPRFPTPL HGHVG---RG AFSDVYEPAE DTFLLLDALE
R. aegyptiacus   ---------- MAAPRFPTPL HGHVG---RG TFSDVYEPAE DTFLLLDALE
B. taurus        ---------- MAAPSFPTPL HGHVG---RG DFSDVYEPAE DTFLLLDALE
E. caballus      ---------- MAALGLPTPL HGHVG---RG AFSDVYEPAE DTFLLLDALE
M. musculus      ---------- MAAPSVPTPL YGHVG---RG AFRDVYEPAE DTFLLLDALE
H. glaber        ---------- MAARSFPTPL HGHVG---RG EFSDVYEPAE DTFLLLDALE
```

```
H. sapiens       ---------- MAGENFATPF HGHVG---RG AFSDVYEPAE DTFLLLDALE
M. domestica     -----MAARC RTPPRFPTPL HRHVG---QG AFREVYEPAE DTFLLLDALE

                 101                                               150
M. rufoniger     XXXAELMQVE LCLEVGSGSG VVSXLLAFMI GPQALYMCTD INPKAAAFTL
M. davidii       ---------- ---------- --------MI GPQALYLCTD INPEAAACTL
M. brandtii      ---------- ---------- --------MI GPQALYMCTD INPKAAACTL
M. lucifugus     RPRGPNRKVE LCLEVGSGSG VVSTFLASMI GPQALYMCTD INPKAAACTL
E. fuscus        AAAAELMRVE LCLEVGSGSG VVSAFLASMI GPRALYVCTD INPNAAACTL
P. alecto        AAVTEFKGVE ICLEVGSGSG VVSAFLATMI GPQVLYMCTD INPNAAVCTL
P. vampyrus      AAVTEFKGVE ICLEVGSGSG VVSAFLATMI GPQVLYMCTD INPNAAVCTL
R. aegyptiacus   AAVAELKGVE ICLEVGSGSG VVSAFLATMI GPQALYMCTD INPNAVACTL
B. taurus        AAAAELTGVE ICLEVGSGSG VVSAFLASVI GPQALYMCTD VNPEAAACTL
E. caballus      AAAAEFTGVE ICLEVGSGSG VVSAFLASMI GPQALYMCTD INPEAAACTL
M. musculus      AAAAELAGVE ICLEVGAGSG VVSAFLASMI GPRALYMCTD INPEAAACTL
H. glaber        AAAPELAGVE ICLEVGSGSG VVSVFLASMI GPQALYMCTD VNPEAAACTL
H. sapiens       AAAAELAGVE ICLEVGSGSG VVSAFLASMI GPQALYMCTD INPEAAACTL
M. domestica     AAAAELMGVE ICLEIGSGSG VVSAFLASII GPQALYMCTD INPKAADCTL

                 151                                               200
M. rufoniger     EIACCNRVHV HPVTTDLVKG LLPRLKEKVD LLVFNPPYVV TLPEE-----
M. davidii       ETARCNRVHV HPVITDLVKG LLPRLKEKVD LLVFNPPYVV TPPEE-----
M. brandtii      ETARCNRVHV HPVITDLVKG LLPRLKEKVD LLVFNPPYVV TPPEEDIMRQ
M. lucifugus     ETARCNRVHV HPVITDLVKG LLPRLKEKVD LLVFNPPYVV TPPEE-----
E. fuscus        ETARCNKVHV QPVITDLVKG LLPRLKEKVD LLVFNPPYVV TPPEE-----
P. alecto        ETARCNKVHI QPIITDLVNG LLPRLKEKVD LLVFNPPYVV TPPEE-----
P. vampyrus      ETARCNKVHI QPIITDLVNG LLPRLKEKVD LLVFNPPYVV TPPEE-----
R. aegyptiacus   ETARCNKVHI QPIITDLVNG LLPRLKEKVD LLVFNPPYVV TPPEE-----
B. taurus        ETARCNKVHI QPIITDLVKG LLPRLKESVD LLVFNPPYVV TPPEE-----
E. caballus      ETARCNSVHI QPVITDLVKG LLPRLKGNVD LLVFNPPYVV TPPEE-----
M. musculus      ETARCNRVHV QPVITDLVHG LLPRLKGKVD LLVFNPPYVV TPPEE-----
```

```
H. glaber        ETAHCNRVHI QPVITDLVKG LLPRLKEKVD LLVFNPPYVV TPPEE-----
H. sapiens       ETARCNKVHI QPVITDLVKG LLPRLTEKVD LLVFNPPYVV TPPQE-----
M. domestica     ETALCNKVHI QPIITDLAKG LLPRLFSKVD LLVFNPPYVV TPSEE-----

                 201                                                250
M. rufoniger     ---------- ---------- ----VGSHGT EAAWXGGRNG REVMDRFFPL
M. davidii       ---------- ---------- ----VGSHGI EAAWAGGRNG REVMDRFFPL
M. brandtii      WVQEKKEIMK IRNLEVISGR HNIPVGSHGI EAAWAGGRNG REVMDRFFPL
M. lucifugus     ---------- ---------- ----VGSHGI EAAWAGGRNG REVMDRFFPL
E. fuscus        ---------- ---------- ----VGSHGI EAAWAGGRNG REVMDRFFPL
P. alecto        ---------- ---------- ----VGSHGI EAAWAGGRNG REVMDRFFPL
P. vampyrus      ---------- ---------- ----VGSHGI EAAWAGGRNG REVMDRFFPL
R. aegyptiacus   ---------- ---------- ----VGSHGI EAAWAGGRNG REVMDRFFPL
B. taurus        ---------- ---------- ----VGSRGI QAAWAGGRNG REVIDRFLPL
E. caballus      ---------- ---------- ----VGSHGI EAAWAGGRNG REVMDRFFPL
M. musculus      ---------- ---------- ----VGSRGI EAAWAGGRNG REVMDRFFPL
H. glaber        ---------- ---------- ----VGSHGI EAAWAGGRNG REVMDRFFPL
H. sapiens       ---------- ---------- ----VGSHGI EAAWAGGRNG REVMDRFFPL
M. domestica     ---------- ---------- ----VGSHGI EAAWAGGRNG REVMDRFFPL

                 251                                                300
M. rufoniger     VPDLFSPRGL LYLVTIKENN PEDILETMKV RGLQGTTALS RRAGQEILSV
M. davidii       VPDLLSPRGL FYLVTIKEND PEDILETMKV RGLQGTTALS RRAGQELLSV
M. brandtii      VPDLLSPRGL FYLVTIKENN PEDILETMKV RGLQGTIALS RRAGQEILSV
M. lucifugus     VPDLLSPRGL FYLVTIKENN PEDILETMKV RGLQGTTALS RRAGQELLSV
E. fuscus        VPDLLSPRGV FYLVTIKENN PEDILETMKV KGLQGTTALS RRAGQEVLSV
P. alecto        APDLLSPRGS FYLVTIKENH PEEILKTMKM KGLQGTAVLC RQAGQETLSV
P. vampyrus      APDLLSPRGS FYLVTIKENH PEEILKTMKI KGLQGTAVLC RQAGQETLSV
R. aegyptiacus   ALDLLSPRGS FYLVTIKENH PEEILKTMKM KGLQGTTALC RQAGQETLSV
B. taurus        APDLLSPRGL FYLVTIKENN PEEILKIMKT KGLQGTTVLS RQAGQEMLSV
E. caballus      VPDLLSPRGL FYLVTIKENN PEEILKTMKT KGLQGTTALS RQAGQELLSI
```

M. musculus	APELLSPRGL	FYLVTVKENN	PEEIFKTMKT	RGLQGTTALC	RQAGQEALSV
H. glaber	ASDLLSPRGL	FYLVTIKENN	PEEILKTLKT	KGLHGTTALS	RQAGQEILSV
H. sapiens	VPDLLSPRGL	FYLVTIKENN	PEEILKIMKT	KGLQGTTALS	RQAGQETLSV
M. domestica	AADLLSREGF	FYLVAIKENN	PDEIMETMKK	YGLHGITVLS	RQAGGETLSV

	301	315
M. rufoniger	LMFTKFSHSV	CSTLY
M. davidii	LRFTKSSHSV	CSTLY
M. brandtii	LRFTKSSHSV	CSTLY
M. lucifugus	LRFTKSSHSV	CSTLY
E. fuscus	LRFTKSSHSV	CSTLQ
P. alecto	LKFTKS----	-----
P. vampyrus	LKFTKS----	-----
R. aegyptiacus	LKFTKS----	-----
B. taurus	LKFTKS----	-----
E. caballus	LKFTRSSHTG	GAP--
M. musculus	LRFSKS----	-----
H. glaber	LKFTKC----	-----
H. sapiens	LKFTKS----	-----
M. domestica	LKFNKC----	-----
